# Cell-specific gene expression in *Anabaena variabilis* grown phototrophically, mixotrophically, and heterotrophically

**DOI:** 10.1186/1471-2164-14-759

**Published:** 2013-11-05

**Authors:** Jeong-Jin Park, Sigal Lechno-Yossef, Coleman Peter Wolk, Claire Vieille

**Affiliations:** Great Lakes Bioenergy Research Center, Michigan State University, East Lansing, MI 48824 USA; Department of Microbiology & Molecular Genetics, Michigan State University, East Lansing, MI 48824 USA; MSU-DOE Plant Research Laboratory, Michigan State University, East Lansing, MI 48824 USA; Department of Plant Biology, Michigan State University, East Lansing, MI 48824 USA; Department of Biochemistry & Molecular Biology, Michigan State University, East Lansing, MI 48824 USA; Present address: Institute of Biological Chemistry, Washington State University, Pullman, WA 99164 USA

**Keywords:** *Anabaena variabilis*, Amino acid biosynthesis, Vegetative cell, Heterocyst, Transcript levels, Microarray

## Abstract

**Background:**

When the filamentous cyanobacterium *Anabaena variabilis* grows aerobically without combined nitrogen, some vegetative cells differentiate into N_2_-fixing heterocysts, while the other vegetative cells perform photosynthesis. Microarrays of sequences within protein-encoding genes were probed with RNA purified from extracts of vegetative cells, from isolated heterocysts, and from whole filaments to investigate transcript levels, and carbon and energy metabolism, in vegetative cells and heterocysts in phototrophic, mixotrophic, and heterotrophic cultures.

**Results:**

Heterocysts represent only 5% to 10% of cells in the filaments. Accordingly, levels of specific transcripts in vegetative cells were with few exceptions very close to those in whole filaments and, also with few exceptions (e.g., *nif1* transcripts), levels of specific transcripts in heterocysts had little effect on the overall level of those transcripts in filaments. In phototrophic, mixotrophic, and heterotrophic growth conditions, respectively, 845, 649, and 846 genes showed more than 2-fold difference (p < 0.01) in transcript levels between vegetative cells and heterocysts. Principal component analysis showed that the culture conditions tested affected transcript patterns strongly in vegetative cells but much less in heterocysts. Transcript levels of the genes involved in phycobilisome assembly, photosynthesis, and CO_2_ assimilation were high in vegetative cells in phototrophic conditions, and decreased when fructose was provided. Our results suggest that Gln, Glu, Ser, Gly, Cys, Thr, and Pro can be actively produced in heterocysts. Whether other protein amino acids are synthesized in heterocysts is unclear. Two possible components of a sucrose transporter were identified that were upregulated in heterocysts in two growth conditions. We consider it likely that genes with unknown function represent a larger fraction of total transcripts in heterocysts than in vegetative cells across growth conditions.

**Conclusions:**

This study provides the first comparison of transcript levels in heterocysts and vegetative cells from heterocyst-bearing filaments of *Anabaena*. Although the data presented do not give a complete picture of metabolism in either type of cell, they provide a metabolic scaffold on which to build future analyses of cell-specific processes and of the interactions of the two types of cells.

**Electronic supplementary material:**

The online version of this article (doi:10.1186/1471-2164-14-759) contains supplementary material, which is available to authorized users.

## Background

*Anabaena variabilis* ATCC 29413 is a well-studied, genetically tractable [1], filamentous cyanobacterium. Its vegetative cells photosynthesize and fix CO_2_. In the presence of oxygen (O_2_) and absence of a source of combined nitrogen, *A. variabilis* fixes atmospheric nitrogen (N_2_) in specialized cells called heterocysts that differentiate from vegetative cells. The semi-regularly spaced heterocysts comprise about 5%-10% of all cells in the filament [2, 3]. Heterocysts are thought to maintain a microoxic interior by three mechanisms: they (i) form a thick envelope of glycolipid and polysaccharide that reduces the rate of entry of O_2_, (ii) respire actively, and (iii) stop producing O_2_[4, 5]. Their microoxic interior permits N_2_ fixation by nitrogenase, a highly O_2_-sensitive enzyme. Hydrogen (H_2_) produced by nitrogenase is largely reassimilated by an uptake hydrogenase, Hup. N_2_ fixed in heterocysts is assimilated through the glutamine synthetase-glutamate synthase (GS-GOGAT) pathway, and glutamine is considered a main nitrogenous product transported to vegetative cells. In exchange, vegetative cells have been thought to transfer sucrose and glutamate to the heterocysts [6–9]. In the light, ferredoxin reduced by photosystem I (PS I) is the likely source of electrons for N_2_ fixation [10], but the metabolic pathway or pathways that transfer electrons to PS I in heterocysts are not known.

Knowledge of cell-specific metabolism in *A. variabilis* and its relatives has been obtained in large part from studies of enzyme assays, the expression of individual genes, and other genetic approaches [4, 11–15]. Numerous studies have focused on regulatory mechanisms governing heterocyst development [14–19] rather than on the metabolism of mature heterocysts. Recent studies have sought a genome-wide understanding of cell-specific metabolism in these cyanobacteria. The first such effort, performed with *A. variabilis*’s close relative, *Anabaena*/*Nostoc* sp. strain PCC 7120 [20] (hereafter called PCC 7120), used microarrays comprising 3-kb DNA fragments covering approximately 90% of the chromosome. The authors compared transcript levels in filaments and in a heterocyst-enriched fraction; the multi-gene features used on the microarrays limited the interpretation of the results. Microarray studies of PCC 7120 [21] and *Nostoc punctiforme*[22] used gene-specific probes and compared gene transcript levels in different growth conditions, but did not attempt to characterize transcript levels in different types of cells. A recent microarray study of PCC 7120 that emphasized growth conditions favoring circadian gene expression [23] characterized transcript levels of several genes in a heterocyst-enhanced fraction (80% heterocysts) versus filaments. RNA-sequencing methods were used to study transcript levels between 0 and 21 h [24] or 0 and 8 h [25] of nitrogen stepdown at the filament level but not in different types of cells. Proteomic analyses of related cyanobacteria [26–28] have unambiguously identified too few proteins (e.g., 377 proteins in [27]) to validate the presence of entire pathways.

^13^C-based metabolic flux analysis, an excellent method for quantifying fluxes in central metabolic pathways [29, 30], has been applied to unicellular cyanobacteria using ^13^C-labeled CO_2_[31]. Provided that one has sufficient knowledge of the amino acid biosynthetic pathways, and other principal pathways, that are active in heterocysts, the metabolism of heterocyst-containing filamentous cyanobacteria can also potentially be studied by metabolic flux analysis by using the ability of *A. variabilis* to assimilate fructose [32, 33]. Very recently, PCC 7120 was shown to grow, albeit exceedingly slowly, when provided with 0.1 or 0.2 M fructose in the dark [34]. It can grow heterotrophically more rapidly when supplemented with fructose transport genes from *A. variabilis*, but still much more slowly than does *A. variabilis*[35]. *A. variabilis* was, therefore, used in our work. As an initial step, we investigated *A. variabilis* cultures grown phototrophically (in the light), mixotrophically (in the light with fructose), and heterotrophically (in the dark with fructose) in the absence of combined nitrogen. These conditions separate the effects of carbon source (CO_2_ vs. fructose) from those of sources of energy and reductant (light vs. fructose) on transcript levels. Our intent is to use gene transcript patterns (i.e., variations of a gene’s transcript levels in different cell types and conditions) identified in this study to model possible metabolic pathways of vegetative cells and mature heterocysts as well as intercellular metabolic networks. Transcript levels were compared in isolated heterocysts, in vegetative cells from heterocyst-bearing filaments (for which there was no precedent), and in whole heterocyst-bearing filaments (to test whether those measurements were consistent). Cell-specific gene transcript levels were analyzed with steady-state cultures, because steady-state cultures would be needed for metabolic flux analysis of N_2_-fixing *A. variabilis* filaments.

## Methods

### Bacterial strain and growth conditions

*A. variabilis* ATCC 29413 was grown in an eightfold dilution of the medium of Allen and Arnon [36, 37] (AA/8). Phototrophic and mixotrophic cultures were grown under continuous illumination by Philips cool white fluorescent lamps, 60–70 μmol photons m^-2^ s^-1^. Mixotrophic cultures were supplemented with 5 mM fructose. Heterotrophic cultures were grown in the dark in the presence of 5 mM fructose. Four hundred-ml phototrophic, mixotrophic, and heterotrophic cultures in 2.8-l Fernbach flasks were inoculated from 50-ml precultures grown in the same conditions. Cultures were inoculated at a concentration of 0.05 μg chlorophyll *a* ml^-1^, and grown on a shaker at 30°C and 140 rpm. Actively growing filaments were harvested after seven days for phototrophic and heterotrophic cultures, and after four days for mixotrophic cultures. Dissolved oxygen was monitored in representative 400-ml cultures using an optical sensor system (Fluorometrix, Stow, MA) with a paper-thin, autoclavable luminescent oxygen sensor taped on the interior bottom surface of the flask, as described in the manufacturer’s instructions.

### Separation of cell type-specific contents for RNA extraction

Cultures (400 ml) were sedimented at 500 × *g* for 5 min at 4°C, resuspended in ~15 ml RNA*later* solution (Ambion, Austin, TX), and stored at -80°C. Once thawed, suspended filaments were sedimented (500 × *g*, 5 min, 4°C), resuspended in 50 ml of N_2_-sparged HP buffer (30 mM Hepes/30 mM Pipes/1.0 mM MgCl_2_, pH 7.2), and washed three times with N_2_-sparged HP buffer containing 10 mM disodium ethylenediaminetetraacetic acid (HP/EDTA). Twenty percent of the suspension was used to extract RNA from whole filaments. The rest was used to isolate and extract heterocysts, by a modification of a published method [2], and to prepare vegetative cell-specific extracts. That method reported a final ratio of ca. 0.01 vegetative cells per heterocyst. The washed filaments were resuspended in 40 ml of HP/EDTA containing 1 mg ml^-1^ lysozyme and were shaken at 30°C for 5 min. The lysozyme-treated suspension was sedimented (500 × *g*, 5 min, 4°C), and the resulting pellet was resuspended in 10 ml of HP buffer in a test tube. The tube was immersed in an ultrasonic cleaning bath (Model 8845–4, Cole-Palmer, Chicago, IL) and was subjected to cavitation for 3 min to destroy a fraction of the vegetative cells. Heterocysts and remaining vegetative cells were sedimented (500 × *g*, 5 min, 4°C), and the clear supernatant fluid (vegetative cell lysate) was saved on ice for extraction of vegetative cell-specific RNA. The sedimented cells were washed twice with HP/EDTA buffer. The washed cells were resuspended in 1 ml of HP/EDTA containing 0.2 mg ml^-1^ lysozyme, shaken at 30°C for 25 min, sedimented (1,000 × *g*, 5 min, 4°C), and the pellet was resuspended in 1 ml of HP buffer. This suspension was immersed in a 12°C sonic bath for 15 min to destroy remaining vegetative cells, and again sedimented (1,000 × *g*, 5 min, 4°C). The supernatant solution was discarded, and the heterocyst-containing pellet was washed three times with HP buffer. Images of the resuspended pellets confirmed a high ratio of heterocysts to fragments of heterocyst envelopes and what may be ruptured remains of vegetative cell or heterocyst protoplasts (not shown).

### RNA extraction

RNA was extracted from whole filaments, isolated heterocysts, and vegetative cell extracts with the RiboPure-Bacteria kit (Ambion) as described [38]. Extracted RNA was purified with an RNeasy Mini kit (Qiagen, Valencia, CA) and eluted in 30 μl of water. RNA preparations were stored at -80°C until use. All RNA extractions were performed on three biological replicates. RNA samples were quantified using a NanoDrop ND-1000 spectrophotometer (NanoDrop Technologies, Wilmington, DE).

### RNA quality and cell-specificity controls

The separate purifications of total RNAs from vegetative cells and from heterocysts from the same culture took close to 5 h. Because of this unavoidable time constraint, our experiments may provide reliable information only for RNAs that are stabilized by Ambion RNA*later* and, perhaps, abundant. The quality of the extracted RNA was tested on an RNA 6000 Nano LabChip (Agilent Technologies, Santa Clara, CA) using a 2100 Bioanalyzer (Agilent Technologies). Reverse transcription followed by quantitative real time-PCR (RT-qPCR) was used to test the cell specificity of RNA extractions. The *rbcL* gene (Ava_3907) was used as a vegetative cell-specific gene and *nifK* (Ava_3930) was used as a heterocyst-specific gene [2, 39]. The RNAse P RNA gene (*rnpB*), constitutively expressed in *A. variabilis*, was used as an internal control for data normalization [40]. In addition, PCR reactions were performed using RNA and cDNA as templates and *rnpB*_F and *rnpB*_R as primers to control for possible contamination of our purified RNA samples with genomic DNA. The gene-specific primers (Additional file 1) were designed using Primer Express 3.0. First-strand cDNA was prepared by reverse transcription using Superscript II reverse transcriptase (Invitrogen, Carlsbad, CA) and a combination of random primers (Invitrogen). 1.5 μl of reverse transcription reaction mixture was used for each RT-qPCR reaction. Each reaction mixture contained 2 μM of each gene-specific primer and 7.5 μl of Power SYBR green PCR master mix (Applied Biosystems, Foster City, CA). RT-qPCR was performed with the three biological replicates on an ABI 7900HT Fast Real-Time PCR System (Applied Biosystems). Relative fold changes in transcript levels were calculated using a standard curve for relative quantification (pools of 1 pg to 250 pg of cDNA were used).

### Microarray experiments

cDNA was synthesized from the twenty-seven RNA samples (three culture conditions, and triplicate RNA extractions from each of whole filaments, vegetative cells, and heterocysts) by the University of Wisconsin-Madison Gene Expression Center. DNA end-labeling, hybridization, scanning, and data normalization were performed by NimbleGen (Reykjavík, Iceland), which provided the final data file. Cy3-labeled cDNAs were hybridized to NimbleGen expression array chips (Product no. A4385-00-01) that represent 5,657 ORFs in the *A. variabilis* genome (GenBank accession no. CP000117) excluding a 49-ORF incision element (GenBank accession no. NC_014000). Each ORF was represented by seventeen 60-mer oligonucleotides. Each oligonucleotide was present four times on the array. The twenty-seven microarray data files were normalized against each other using quantile normalization [41]. Expression array data were analyzed using ArrayStar 3.0 (DNASTAR, Madison, WI). Microarray data have been deposited in the National Center for Biotechnology Information Gene Expression Omnibus database (http://www.ncbi.nlm.nih.gov/geo/, accession number GSE46076).

In this paper, upregulation of a gene in a given cell type means upregulation in comparison to the other cell type in the same condition(s). A gene will be said to be transcribed at background, just above background, very low, low, moderate, high, and very high levels in a particular condition when its normalized transcript level is in the range of ≤150, 151–200, 201–600, 601–2,000, 2,001-6,000, 6,001-20,000, or 20,001-60,000 signal intensity units (SIU after normalization) in that condition, respectively. A distinction between “background” and “just above background” is somewhat arbitrary: some genes in one of these categories may belong in the other.

### Statistical data analyses

Principal component analysis (PCA) was performed in Statistica (version 7.0, StatSoft, Tulsa, OK). Cell types and culture conditions were set as categorical variables and transcript levels were set as continuous variables. Linear modeling of the transcript data in each growth condition was performed in R [42] using the function F_i_ = aV_i_ + bHt_i_ - 1, where F_i_, V_i_, and Ht_i_ represent the means of gene i transcript levels in filaments, vegetative cells, and heterocysts, respectively; a and b are constants that reflect the relative abundance of vegetative cells and heterocysts in the filaments; and -1 is a term that forces the intercept to 0. Calculations of Spearman’s rank correlation coefficients [43], grid searching, and bootstrapping were performed in R. Weighted residuals were calculated using Equation 1, where R_i_ is the weighted residual of gene i, F_i,calc_ = aV_i_ + bHt_i_, and  is the length of the (V_i_, Ht_i_, F_i_) vector in three-dimensional space.1

### Cell extracts and enzyme assays

For enzyme assays of *A. variabilis* grown in phototrophic conditions, cultures were harvested by centrifugation when chlorophyll concentration reached 8 μg/ml, and stored at -80°C. To prepare crude extracts from whole filaments, cells from 200-ml cultures were resuspended in 10 ml lysis buffer (50 mM Tris–HCl, pH 8.4, containing 1 mM phenylmethylsulfonyl fluoride and one protease inhibitor cocktail tablet [complete mini, EDTA-free, Roche Diagnostics, Indianapolis, IN]). Cells were lysed by two passages through a French press maintained at 4°C (4,000 to 5,000 psi). After centrifugation of the whole filament lysate (2,000 × *g*, 15 min, 4°C), the supernatant solution was dialyzed twice against 20 mM Tris–HCl (pH 7.2), with a total dialysis time of 24 h (SpectraPor dialysis tubing, 12,000-14,000 Da cut-off, Spectrum Laboratories, Rancho Dominguez, CA). Dialysis was required to remove phosphates from the lysate. The dialyzed filament lysate was used in enzyme assays. To prepare crude extracts of enriched heterocyst fractions, heterocysts were purified as described for RNA purification. Purified heterocysts were resuspended in 1.5 ml lysis buffer and lysed using zirconia beads in a Mini-BeadBeater (Biospec Products, Bartlesville, OK) on high speed setting (1 min, 4°C). After centrifugation (1,600 × *g*, 10 min, 4°C), the supernatant solution―representing the soluble extract―was concentrated by ultrafiltration, and used for protein and enzyme activity assays. Protein concentrations were determined using the Bio-Rad protein assay kit (Bio-Rad, Richmond, CA), with bovine serum albumin as the standard.

Phosphoserine phosphatase activity was measured at 30°C as described [44], using 13–270 μg protein in each assay. The phosphate released was quantified using the malachite green method [45] on a DU-650 spectrophotometer (Beckman, Fullerton, CA).

## Results

### Concentration of dissolved oxygen in cultures

To avoid potential contaminations, particularly in cultures grown with fructose, cultures were shaken under ambient air, but not bubbled. Dissolved O_2_ was monitored during growth to confirm that cultures were fully aerobic (data not shown). Between inoculation and harvest, the dissolved O_2_ in phototrophic and mixotrophic cultures increased from 6.1 mg l^-1^ just after inoculation to 7.5 mg l^-1^ O_2_ at harvest time (7.5 mg l^-1^ is the O_2_ saturation value at 30°C). The dissolved O_2_ in heterotrophic cultures varied between 6.1 mg l^-1^ and 6.3 mg l^-1^ during the entire growth period.

### Quality and cell-specificity of RNA extractions

Only heterocyst RNAs from phototrophic cultures showed evidence of degradation, with most of the degraded RNA species over 200 nt long (Additional file 2: Figure S1). Because reverse transcription of bacterial RNA used random primers, and because each gene on the microarray was represented by seventeen probes, microarray experiments were nonetheless likely to capture most of the abundant RNAs. RNA extractions from heterocysts of phototrophic cultures, repeated for nine biological replicates, yielded similar degradation results. The samples that looked the least degraded were used for microarray experiments. Heterocyst RNAs from phototrophic cultures show, otherwise, trends in transcript levels very similar to those observed with heterocyst RNAs from mixotrophic and heterotrophic cultures (see Overall microarray assessment section), suggesting that RNA degradation in extracts from phototrophic cultures is not a major limitation in our experiments. PCR reactions using RNA samples as templates never showed a PCR band and always showed a PCR band with cDNA controls (data not shown), indicating that our RNA preparations were devoid of contamination by genomic DNA.

**Figure 1:**
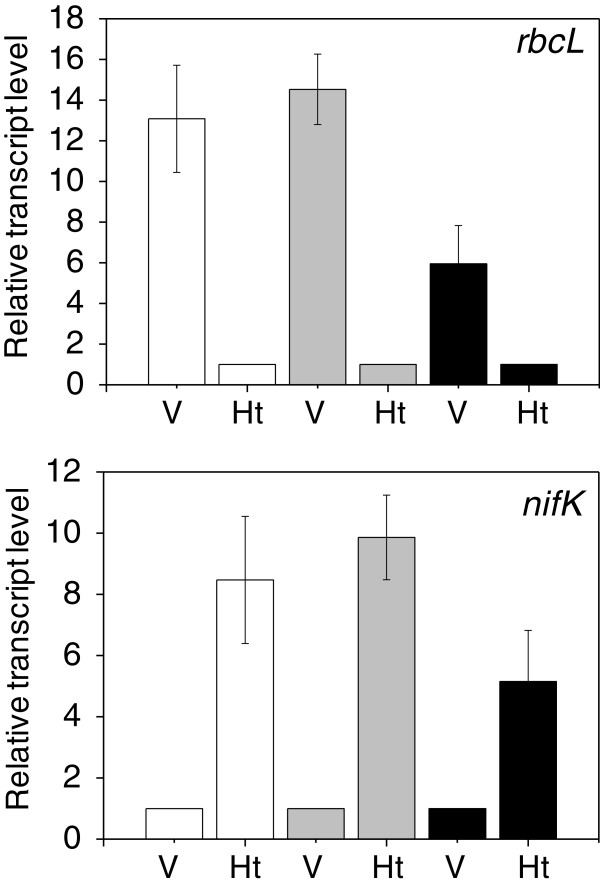
**Verification of cell specificity of RNA extractions by RT-qPCR.** *rbcL* and *nifK* were used as the probes for genes expressed specifically (see text) in vegetative cells (*rbcL*, top panel) and in heterocysts (*nifK*, bottom panel). The internal standard was *rnpB*, which is expressed constitutively in all cells [40]. Culture conditions are shown in white (phototrophic), gray (mixotrophic), and black (heterotrophic). V: RNA extracted from vegetative cells; Ht: RNA extracted from heterocysts. Means and standard deviations are based on three biological replicates. Transcript levels are normalized to 1 in heterocysts (*rbcL*) and in vegetative cells (*nifK*).

The cell specificity of our RNA preparations was tested by RT-qPCR. We chose *nifK* and *rbcL* as cell specificity marker genes because it is well established that under oxic conditions *nifK* is expressed only in heterocysts and *rbcL* is expressed mostly, perhaps only, in vegetative cells [4, 14]. Ct (threshold cycle) values for *rnpB* did not vary by more than 5% between heterocysts and vegetative cells in all three growth conditions (not shown), validating our choice of *rnpB* as a constitutively expressed gene that can be used to normalize the transcript levels of other genes across experiments. The relative *rbcL* signals obtained from heterocyst RNA were only 7.6% and 6.9% of those obtained from vegetative cell RNA in phototrophic and mixotrophic cultures, respectively (Figure 1). In contrast, the relative *nifK* signals obtained from vegetative cell RNA were only 11.8% and 10.1% of those obtained from heterocyst RNA in phototrophic and mixotrophic cultures, respectively. A conservative interpretation of these results is that heterocyst RNA preparations were over 9% and over 93% cell-specific for phototrophic and mixotrophic conditions, respectively. Vegetative cell RNA preparations were over 88% and 89% cell-specific for phototrophic and mixotrophic conditions, respectively. With heterotrophic cultures, the cell specificity of heterocyst RNA and vegetative cell RNA preparations never appeared to be above 83%, even though RNA extractions were repeated eight times, each time making the first lysis step gentler and the last lysis step harsher to better separate RNA from the two cell types.

If a transcript is more abundant in filaments than in vegetative cells, and yet this transcript is only modestly more abundant―or even less abundant―in heterocysts than in filaments, the heterocyst level of that transcript is likely under-represented in our experiments (examples, including nitrogenase [*nif1*] transcripts, are presented below). When transcript levels in whole filaments are consistent with transcript levels in vegetative cells and heterocysts, and in particular when specific genes are transcribed at high levels across cell types and growth conditions, and in the absence of contradictory information, we consider those genes―or whole pathways―active in heterocysts. On the other hand, transcript levels only slightly above background level in heterocysts will not be considered as evidence that genes or intact pathways are active in heterocysts, even though they may be. We are trying to be conservative in our interpretations in this first effort to use microarray data to identify active pathways in vegetative cells and heterocysts of N_2_-fixing filaments, especially because the importance of major enzymatic pathways (including nitrogen fixation, the processing of sucrose by invertase, the oxidative pentose phosphate cycle, and cytochrome oxidase activity) might otherwise be misinterpreted.

### Overall microarray assessment

The experimental metrics report provided by NimbleGen (not shown) gives summary statistics that can be used to help identify potential problems during hybridization. All metrics for the twenty seven microarray experiments were within the manufacturer’s suggested ranges.

The normalized microarray data are shown in Additional file 3. The coefficients of determination (R^2^ values) between the twenty seven experiments were calculated to quantify experimental variability between biological replicates (Additional file 4). Reproducibility was high for biological replicates of the same experiment, as indicated by R^2^ values ranging between 0.857 and 0.998. The R^2^ values between microarrays using heterocyst RNAs isolated from different culture conditions were also high, between 0.827 and 0.976. These results also include the experiments with the partially degraded heterocyst RNAs extracted from phototrophic cultures, suggesting that partial degradation of the RNA has only a minor effect on overall hybridization results. The R^2^ values between microarrays using vegetative cell RNAs and whole filament RNAs isolated from the same culture types also were high, between 0.876 and 0.994, reflecting the fact that filaments comprise mostly vegetative cells. In contrast, microarray results varied more when comparing vegetative cell RNAs extracted from different types of cultures (R^2^ values between 0.542 and 0.817) or when comparing heterocyst and vegetative cell RNAs from the same cultures (R^2^ values between 0.400 and 0.871). These results make sense based on the respective metabolic functions of vegetative cells and heterocysts (see explanation below).

**Figure 2:**
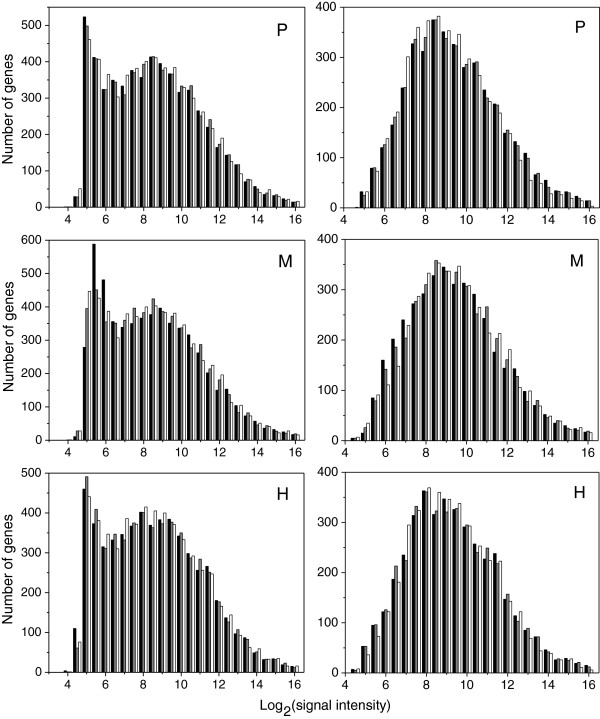
**Histograms of the log**_**2**_ **values of the normalized average signal intensities for all microarray experiments.** (P): Phototrophic cultures; (M): mixotrophic cultures; and (H): heterotrophic cultures. Black: signal distribution in whole filaments; gray: signal distribution in vegetative cells; and white: signal distribution in heterocysts. Left panels: all genes; right panels: reduced sets of genes (see Additional files 5 and 6; P: 3,949 genes; M: 3,885 genes, and H: 3,933 genes).

In all growth conditions and for each cell type, signal intensities were not normally distributed (Figure 2, left panels). A high number of genes with low intensity signals (log_2_ [intensity] below 7.0) is found across all experiments, independent of cell type and culture condition, and may correspond to genes whose RNA is disproportionately labile. The proportion of genes with low signal intensity in the heterocysts of phototrophic cultures is not higher than it is in vegetative cells or whole filaments in the same culture conditions (Figure 2, top left panel). This observation suggests that the poorer quality of the RNA extracted from the heterocysts of phototrophic cultures did not substantially bias the results.

Microarray experiments with RNA from whole filaments were used to validate the results of the experiments performed with cell-specific RNA. In N_2_-fixing *A. variabilis* filaments, transcript levels of any gene, i, should be consistent with the equation, F_i_ = aV_i_ + bHt_i_. Assuming that heterocysts and vegetative cells contain similar amounts of RNA and assuming that RNA is extracted with the same yield from whole filaments, vegetative cells, and heterocysts, a + b should equal 1, with the a-value ranging between 0.9 and 1 and the b-value ranging between 0 and 0.1. Linear modeling was applied to reduced data sets (Additional file 5), where genes that showed average transcript levels below 128 across experiments and genes with high variability between biological replicates were removed (see Additional file 6 for details). The values of a and b were determined for the three growth conditions (Additional file 6). With the exceptions that the a-value was above 1 in phototrophic and heterotrophic conditions and the b-value was below 0 in heterotrophic conditions, the calculated values for a and b were generally in the ranges expected from the frequency of heterocysts in filaments (i.e., a ~ 0.92 and b ~ 0.08). Although we do not know whether heterocysts and vegetative cells have the same amounts of mRNA, equal amounts of cDNA were used in all hybridization experiments, possibly biasing the values of a and b during linear modeling. Our results remain consistent with the idea that for most genes the transcript level of a gene in heterocysts contributes little to the transcript level of this gene in whole filaments. Thus for most genes, transcript levels in whole filaments closely approximate transcript levels in vegetative cells.

In phototrophic and mixotrophic conditions, few genes in the reduced data set behaved as outliers, with transcript level data that did not closely conform to the equation F_i_ = aV_i_ + bHt_i_. (Outliers are not discussed for heterotrophic conditions because the value of b was not reliable: see Additional file 6). Deviation from the linear equation suggests that RNA is degraded in one type of cell or the other. The most conspicuous outliers (i.e., the points farthest from the plane defined by F = aV + bH_t_) were identified in each growth condition by calculating weighted residuals as a proportion of each gene’s transcript level using equation 1 (Additional file 6). Two sets of outlier genes in phototrophic conditions warrant mention. The *nif1* genes, *nifB, S*, *U*, *H*, *D*, *K*, *E*, *N*, *X,* and *W* (Ava_3912, Ava_3914-3917, Ava_3930, Ava_3932-3934, and Ava_3937, respectively) were the 3^rd^ to 12^th^ outliers for which F_i_ > > aV_i_ + bHt_i_. The transcript levels of *nif1* genes and of related maturation genes should be strongly upregulated in heterocysts compared to vegetative cells [2, 39, 46], and the signal intensities for these genes should be ca. 10-fold lower in whole filaments than in heterocysts. Instead―especially in phototrophic conditions―signal intensities for *nif1* genes were nearly always higher in whole filaments than in heterocysts, implying that the signal intensities in heterocysts were at least 10-fold lower than expected. This observation suggests that the *nif1* transcripts are specifically targeted for rapid degradation in heterocysts upon separation of the heterocysts from vegetative cells under aerobic conditions. Transcripts of *nif1* genes may represent a large fraction of the degraded transcripts seen in heterocysts of phototrophic cultures (Additional file 2: Figure S1). These results might be related to the degradation of *nifHDK* transcripts observed in PCC 7120 [47]. Because certain *nif1* transcripts accumulated to up to 44% of the most abundant transcript in heterocysts in these conditions (consistent with the very large amount of protein attributable to Nif in non-denaturing gels of *A. variabilis* heterocysts [2]), the seemingly artificially low transcript levels for *nif1* genes likely caused a factitious increase of transcript levels for all other genes in the heterocysts of phototrophic cultures. Therefore, moderate upregulation (below 5-fold) of genes other than *nif1* in the heterocysts of phototrophic cultures may not be meaningful. Second, five PS II genes (Ava_4121, Ava_0593, Ava_1597, Ava_3553, and Ava_2460, four of them *psbA* genes) are the top two and the top 13^th^ to 15^th^ outliers. These genes have signal intensities in filaments that are 1.6- to 38-fold lower than in vegetative cells. This trend in transcript levels of *psbA* genes is reminiscent of what happens in cyanobacteria subjected to oxidative damage (see Targeted analysis-Photosystems).

### General analysis of microarray results

The only other use made of the reduced data sets (Additional files 5 and 6) was to highlight the differences in transcript levels between vegetative cells and heterocysts in the different growth conditions using volcano plots (Additional file 2: Figure S2). P values for those plots were calculated using two-tailed t-tests with unequal variances. Two hundred eighty, 144, and 545 genes were significantly upregulated (over 2-fold difference with p < 0.01) in vegetative cells in phototrophic, mixotrophic, and heterotrophic cultures, respectively. Of these genes, 22.5% to 24.3% had unknown products. Five hundred sixty five, 505, and 301 genes were significantly upregulated (over 2-fold difference with p < 0.01) in heterocysts in phototrophic, mixotrophic, and heterotrophic cultures, respectively. Of these, 36.8% (in phototrophic conditions) to 46.2% (in heterotrophic conditions) were genes with unknown products. Of the genes with unknown products that were upregulated in one type of cell versus the other, 77%, 86%, and 51% were upregulated in the heterocysts in phototrophic, mixotrophic, and heterotrophic conditions, respectively. In summary, although transcript levels in vegetative cells and heterocysts are highly correlated (Additional file 6), many genes were significantly upregulated in one cell type versus the other in each growth condition.

**Figure 3:**
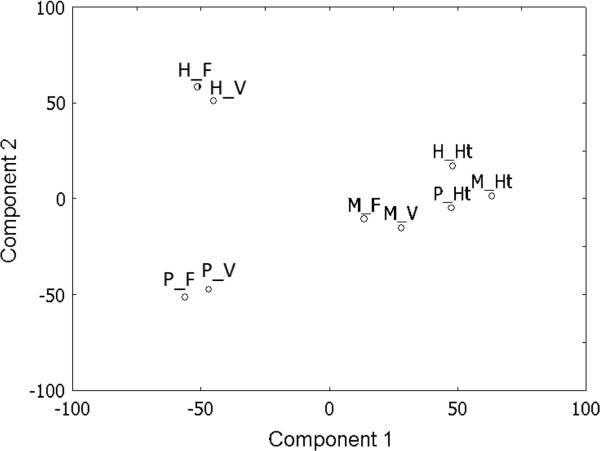
**Principal component analysis of gene expression patterns in different cell types and different growth conditions.** Component 1 is plotted versus component 2. PCA was performed using the entire normalized data set of 5,657 genes. F: whole filaments; H: heterotrophic conditions; Ht: heterocysts; M: mixotrophic conditions; P: phototrophic conditions; and V: vegetative cells.

PCA was used to determine how gene transcript patterns relate to cell type and culture conditions. In the three culture conditions, principal components for the whole filament were close to those for vegetative cells, but not to those for heterocysts (Figure 3), agreeing with the fact that vegetative cells typically represent 90% to 95% of total cells in the filaments. Principal components for vegetative cells varied significantly between growth conditions. These results agree with the fact that vegetative cells are responsible for uptake of carbon and energy, and for the generation of reductant, and with the fact that carbon, energy, and reductant are the parameters that vary between growth conditions. In contrast, principal components for heterocysts varied little between growth conditions. Heterocysts are consistently responsible for nitrogen fixation. The lack of change of principal components for heterocysts in heterotrophic conditions suggests that access to light is not among the top determinants of transcript levels in heterocysts. The fact that heterocyst-specific PCA results (Figure 3) and volcano plots from phototrophic cultures (Additional file 2: Figure S2) are not clearly distinguishable from those of mixotrophic and heterotrophic cultures helps to validate our decision to use seemingly partially degraded heterocyst RNAs from phototrophic cultures for our microarray studies.

### Functional categorization of microarray data

**Figure 4:**
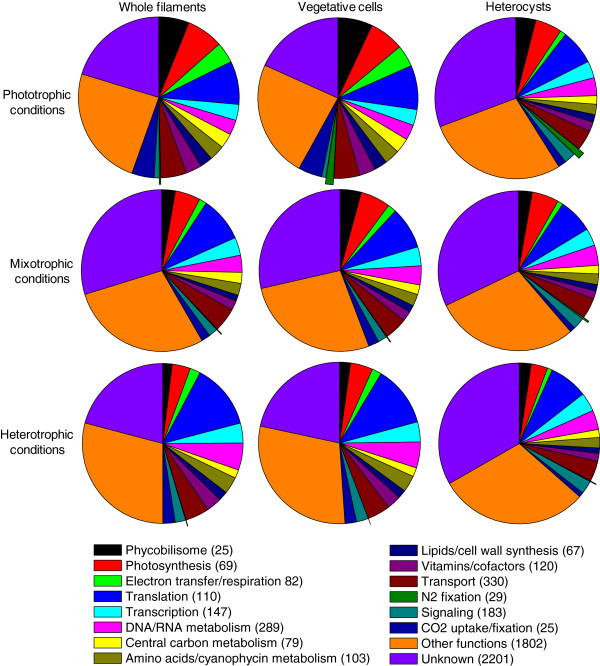
**Distribution of gene transcript levels in functional categories.** Transcript levels of the genes participating in different pathways are represented as percent of total genome transcripts in each experiment. The number of genes in each functional category is given in parentheses. The N_2_-fixation genes are represented by a wedge with an enlarged radius.

To determine which pathways are upregulated in the different growth conditions and in the different cell types, the 5,657 ORFs represented in the microarrays were classified in sixteen functional categories (Additional file 3). Fourteen categories were based on the Kyoto Encyclopedia of Genes and Genomes (KEGG) pathway database [48], Blastp searches [49], and previous publications of gene functions. ORFs annotated only with a protein domain name were arbitrarily included in the Other functions category and those annotated as hypothetical proteins or proteins of unknown function were arbitrarily grouped in the Unknown category. The Other and Unknown categories contained 1,802 and 2,201 genes, respectively (Additional file 3). Since filaments consist mostly of vegetative cells, distribution of transcript levels per functional category was highly similar in whole filaments and vegetative cells in each growth condition tested, as expected (Figure 4). Because sources of carbon and energy are the parameters that vary between growth conditions, the pathways that were upregulated in vegetative cells (and whole filaments) varied widely from one growth condition to another. In contrast, distribution of transcript levels in terms of functional category varied little in heterocysts across growth conditions, agreeing with the fact that heterocysts perform the same main metabolic function, N_2_ fixation across the three growth conditions (Figure 4). These results agree with our PCA results.

The genes involved in phycobilisome assembly, photosynthesis, and CO_2_ uptake/fixation were clearly upregulated in vegetative cells in phototrophic conditions. Transcript levels of these genes decreased in mixotrophic conditions, and even further in heterotrophic conditions, where all carbon and reducing power come from fructose. Genes involved in electron transfer and respiration were unexpectedly down-regulated in heterocysts across growth conditions. This observation does not support the common understanding that heterocysts actively respire [50, 51] as a way to decrease intracellular O_2_ concentrations [4, 52, 53]. However, this appears to be another instance in which, at least under heterotrophic conditions and for several oxidase subunits, the transcript level in heterocysts is likely under-represented.

### Targeted analysis

In this section our results will be described in terms of individual pathways, with a particular focus on pathways that we plan to study later by metabolic flux analysis (e.g., central carbon metabolism as well as nitrogen fixation and amino acid synthesis).

#### Nitrogen fixation

Of the three sets of nitrogenase genes (*nif1*, *nif2*, and *vnf*) present in *A. variabilis,* only the *nif1* cluster is expected to be transcribed in aerobic N_2_-fixing cultures of *A. variabilis* grown in the presence of Mo [46, 54–56]. Indeed, with the exception of *nifH2* (Ava_4247) whose transcript level reached 1.5% of the most abundant transcript in the vegetative cells of phototrophic cultures, *nif2* genes had background to very low transcript levels in all experiments (Additional file 7). Transcript levels of the *vnf* genes were even lower than those of the *nif2* genes in all experiments. As expected, every gene in the *nif1* cluster was strongly upregulated in heterocysts of phototrophic and mixotrophic cultures (Additional file 7). In phototrophic conditions the upregulation of the *nif1* genes in heterocysts ranged between 5.3-fold (*nifU*, Ava_3915) and 22-fold (*nifB*, Ava_3912), all with p < 0.0001. The *nifH*, *nifD*, and *nifK* signals in heterocysts reached 44%, 39%, and 15% of the strongest signal in these cells, respectively. Ava_3940, encoding the ferredoxin FdxH1 that is believed to be the primary electron donor to nitrogenase [10], was also upregulated 15-fold in heterocysts of phototrophic cultures (p ~ 0.05).

Transcripts of *nif1* genes are highly upregulated during the late stages of heterocyst differentiation [17, 39] and their products appear to represent a large portion of the soluble protein of anoxically isolated heterocysts [2]. Nonetheless, transcripts of N_2_ fixation genes represented only 1.4% of total transcripts in heterocysts in phototrophic conditions, reflecting a likely 10-fold or greater underestimate of transcript levels of *nif1* genes in these cells. It remains possible that RNA*later* has difficulty traversing the barrier represented by the heterocyst envelope, so that *nif1* transcripts (and likely other transcripts; see below) were extensively degraded. Because of microarray normalization, highly stable transcripts are likely over-represented in the heterocyst transcriptome.

The *nif1* genes were also upregulated in heterocysts in mixotrophic conditions―between 1.6-fold (*nifU*) and 6.9-fold (*nifS,* Ava_3914), with p values between 0.01 and 0.05―but not to the same extent as in phototrophic conditions. In heterotrophic conditions the *nif1* genes were, at most, moderately upregulated in heterocysts, with p values never under 0.01, and the *nifD* transcript reached only 2.3% of the highest heterocyst transcript. Several reasons could contribute, exclusively or in combination, to the low *nif1* transcript levels in heterotrophic cultures: these cultures are energy-deprived compared to cultures grown in light, the *nif1* RNAs might be partially degraded in our RNA preparations, and nitrogenase might be particularly stable in these conditions.

#### Amino acid biosynthesis

**Figure 5:**
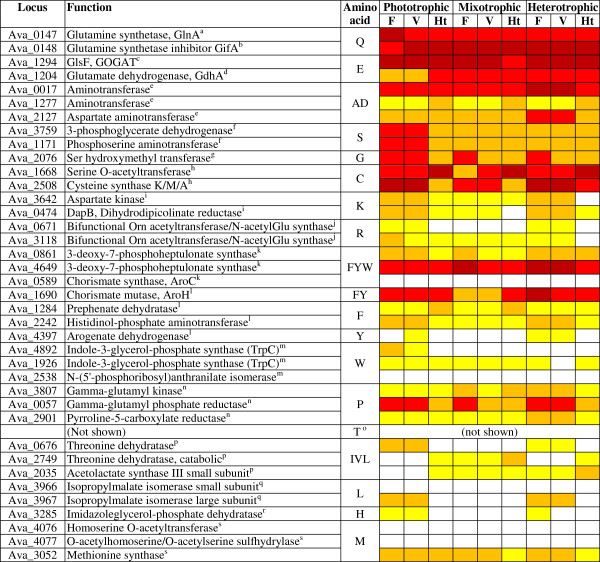
**Transcripts of amino acid biosynthetic genes.** White, yellow, orange, red, and crimson represent transcript levels in the ranges ≤ 200, 201–600, 601–2000, 2001–6000, and ≥ 6001 SIU, respectively. Transcript levels of all genes involved in amino acid biosynthesis are shown in Additional file 7. Legends for superscripts “a” to “s” are in Additional file 8.

Whereas synthesis of Gln and Glu in N_2_-fixing filaments has been the focus of many studies because they are responsible for ammonia assimilation after N_2_ fixation, where and how the other amino acids are synthesized have not been looked at in much detail. Starting from the amino acid biosynthetic genes identified in *A. variabilis* in the KEGG database [57, 58], Blastp comparisons were used to verify all annotations and to identify which pathways are active. Not all pathways and genes could be identified with certainty, in particular enzymes involved in amination (i.e., Asn synthetase) and transamination reactions. The pathways shown in Figure 5 (extra comments in Additional file 8) and Additional file 7 represent the predominant amino acid biosynthetic pathways in *A. variabilis* based on the KEGG database, pathways that are common in the bacterial world [59, 60], known amino acid synthesis pathways in cyanobacteria, and pathways supported by earlier isotope labeling studies.

Amino acid biosynthetic genes were typically either upregulated in vegetative cells or transcribed at similar levels in the two cell types (Figure 5). Only select genes appeared upregulated in heterocysts (e.g., Ava_1668, with p ≤ 0.05) (Figure 5). A few instances were found in which multiple genes encoding isozymes showed different transcript patterns. Most amino acid biosynthetic genes are not organized in operons in *A. variabilis*, so one gene can be transcribed at a very low level, while all other genes in the pathway are transcribed at significant levels. Several genes showed background level transcripts across experiments, possibly due to mRNA instability, making it impossible to predict in which cell type these genes are transcribed (Figure 5). Using a signal intensity cutoff of 200 as the minimum, transcript levels in heterocysts plus the phosphoserine phosphatase activity detected in the crude extracts of heterocysts of phototrophic cultures (footnote f of Figure 5) suggest that Gln, Glu, Ser, Gly, Cys, Thr, and Pro are actively produced in heterocysts. Whether or not the other protein amino acids are actively synthesized in heterocysts is unclear based on our data, because of genes not identified or of transcript levels below 200 SIU for some genes in a given pathway (Figure 5).

**Figure 6:**
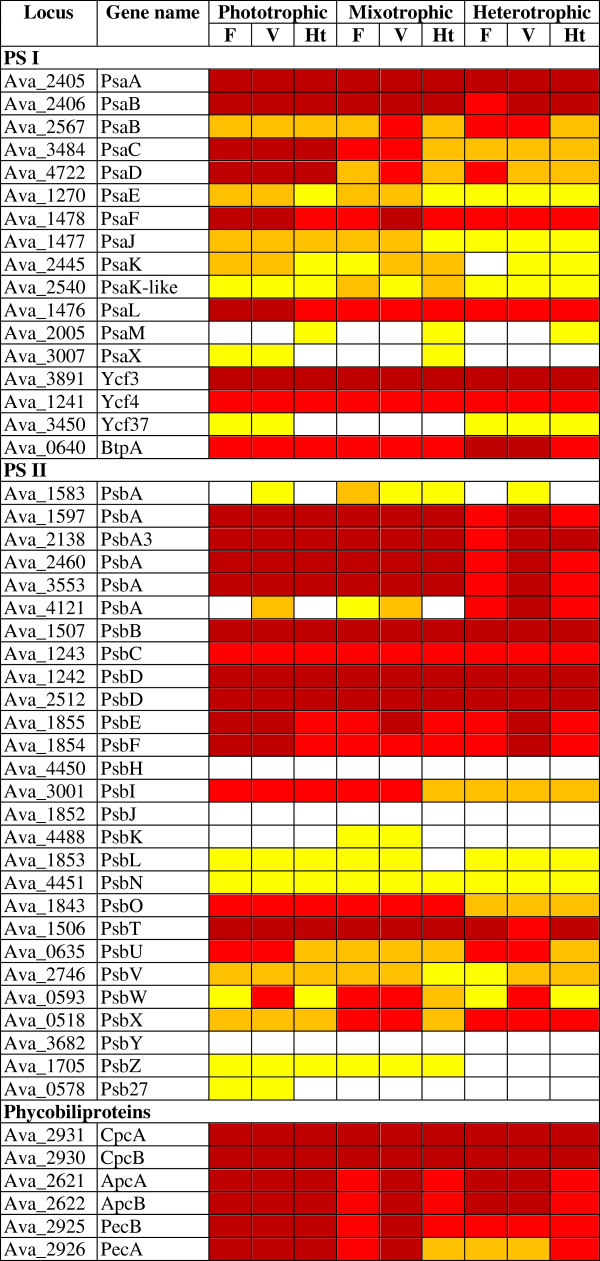
**Transcripts of genes encoding PS I, PS II, and phycobiliproteins.** Colors are as in Figure 5.

The breakdown of phycobiliproteins in heterocysts has been studied as a possible major source of amino acids for de novo protein synthesis in heterocysts [61, 62]. All phycobiliprotein-encoding genes were still transcribed at significant levels in the heterocysts of phototrophic cultures (Figure 6). *nblA* (Ava_3383), encoding a protein required for the breakdown of phycobiliproteins was upregulated 2.2-fold (p < 0.01) in the heterocysts of phototrophic cultures, but not in other growth conditions. The alanine dehydrogenase gene Ava_0176, required for the breakdown of phycobiliproteins in *Synechococcus* PCC 7942 [63], was downregulated in heterocysts across growth conditions. These collective results suggest that while the breakdown of phycobiliproteins may contribute much of the amino acids needed during heterocyst differentiation, it may contribute little to protein repair and protein de novo synthesis in mature heterocysts. This conclusion is consistent with labeling experiments that showed that newly forming and mature heterocysts of *A. oscillarioides* incorporated significant levels of ^13^C and ^15^N in cultures grown with NaH^13^CO_3_ and ^15^N_2_[64].

#### Transport of amino acids and other metabolites

Three PCC 7120 ATP-binding cassette (ABC) transporters specific for amino acids have been characterized: two neutral amino acid transporters, N-I and N-II, and a basic amino acid transporter, Bgt. Both N-I (composed of NatABCDE) and N-II (composed of NatFGH and BgtA) contribute to diazotrophic growth (Gln is a substrate for both transporters), but Bgt is not required [65–68]. In our work, *natA* and *natG* had background transcript levels in all conditions. The other N-I and N-II genes had transcript levels reaching 0.25% to 5.7% of the highest transcripts (Additional file 7). The single Bgt-specific gene, *bgtB*, had background-level signal intensities across all experiments (Additional file 7). Recent studies suggest that the septosome (formerly microplasmodesmata) allows passive diffusion of molecules up to 623 Da, and that SepJ, FraC, and FraD are involved in transport via the septosome [69–71]. While the *sepJ* and *fraD* transcripts were detected across all experiments (*sepJ*) and in vegetative cells only (*fraD*), the *fraC* transcript was barely detectable in any condition (Additional file 7). Background transcript levels for some of these genes (e.g., *natA*, *natG*, and *fraC*) might reflect low abundance and/or high stability of these proteins in vivo.

#### Photosystems

In heterocyst-forming cyanobacteria, vegetative cells express both PS I and PS II; they use water as the electron donor and produce O_2_. Heterocysts use PS I to generate ATP [72]. Transcript levels of eighteen PS I-related genes (Figure 6 and Additional file 7) were compared in all experiments. *psaA-E*, *psaJ-K*, and *ycf37* showed no significant difference in signal intensity between heterocysts and vegetative cells across growth conditions. *psaF*, *L*, and *X*, as well as *ycf3, ycf4*, and *bptA* were upregulated 2- to 3-fold in vegetative cells (p < 0.01), some of them only in phototrophic conditions. These five genes encode proteins involved in PS I docking (PsaF), PS I oligomerization (PsaL), PS I assembly (Ycf3 and 4), or have an unknown function (BptA). Why some PS I genes are more upregulated than others may relate to a different ratio of ATP to reduced ferredoxin needed for the different metabolic processes in heterocysts and vegetative cells.

PS II―and, in particular, its protein PsbA―is prone to oxidative damage [73, 74]. The main response in cyanobacteria is transcriptional: specific *psbA* genes are upregulated. In steady state conditions, PsbA1 is the most abundant PsbA protein in PS II, and its transcript―typically not upregulated in stress conditions―is the most abundant *psbA* transcript [73]. Accordingly, the transcript level for Ava_2138 (*psbA1*) was the most abundant *psbA* transcript in filaments of phototrophic cultures, with similar levels in vegetative cells. Other *psbA* genes showed higher transcript levels in vegetative cells than in filaments, most conspicuously under heterotrophic conditions (Additional file 7). These results suggest that the cavitation used to lyse vegetative cells causes oxidative stress that upregulates transcript levels of certain *psbA* genes. *psbW* behaves similarly, suggesting that it, too, may be involved in PS II repair.

Heterocysts were long thought to have no PS II and to lack the ability to evolve O_2_[75–77]. Our results (Figure 6 and Additional file 7) support recent proteomic observations that find PS II proteins in heterocysts [26–28, 78]. In particular, four of the six *psbA* genes, *psbB*, *psbC*, and the two *psbD* genes showed similar signal intensity levels in vegetative cells and heterocysts across growth conditions. The transcripts of *psbA3*, *psbD* (Ava_1242), and *psbB* even reached 70% to 92% of the most abundant heterocyst transcripts in phototrophic and mixotrophic cultures. This observation suggests that *psb* transcripts are not just inherited from a pre-heterocyst cell, but are actively produced in mature heterocysts as well. Other *psb* genes tended to have higher signal intensities in vegetative cells, but—with the exception of *psbH*, *psbJ,* and *psbY* that had background transcript levels in all experiments—were usually still transcribed in heterocysts. PsbO, U, and V, which stabilize the O_2_-evolving complex [79], had transcripts upregulated between 1.9- and 2.7-fold (p < 0.02) in vegetative cells of phototrophic cultures, but were still transcribed in heterocysts across growth conditions (Figure 6).

#### Photosynthetic pigments

Fewer phycobilisomes, the main light-harvesting complexes for PS II in the vegetative cells [75], could account for a diminution of O_2_-evolving activity of PS II in heterocysts. Spectrophotometric studies suggest that heterocysts contain almost no allophycocyanin, that their low phycocyanin content varies with light intensity, and that their phycobiliproteins may transfer light energy to PS I [76, 80]. Transcripts for the *A. variabilis* phycocyanin genes, *cpcAB*, were found to be over 20-fold more abundant in vegetative cells than in heterocysts 16 h after nitrogen step-down [81]. Transcript levels for all phycobiliprotein genes in heterocysts of phototrophic cultures were much higher in our experiments than expected from previous studies. Signal intensities for *cpcAB*, *apcAB*, and *pecAB* in heterocysts were 80%, 33%, and 35% to 45% of those in vegetative cells, respectively (all with p < 0.058) (Figure 6 and Additional file 7). Signal intensities for *cpcAB* remained very high in vegetative cells and heterocysts of mixotrophic cultures, while transcript levels of *apcAB* and *pecAB* decreased over 6-fold in mixotrophic conditions.

Ten chlorophyll biosynthetic genes were upregulated in vegetative cells of phototrophic cultures, although not all in a statistically significant manner (Additional file 7). Ava_4393, encoding one of three coproporphyrinogen oxidases, stood apart, being upregulated 3.8- to 5.1-fold in heterocysts across growth conditions (p ≤ 0.03). This oxidase may participate in heme synthesis. Twelve chlorophyll biosynthetic genes were downregulated at least 2.5-fold in the filaments of mixotrophic cultures compared to phototrophic conditions, with the first dedicated gene in the pathway (Ava_3699, encoding glutamyl-tRNA reductase) downregulated 19-fold.

#### CO_2_ fixation

**Figure 7:**
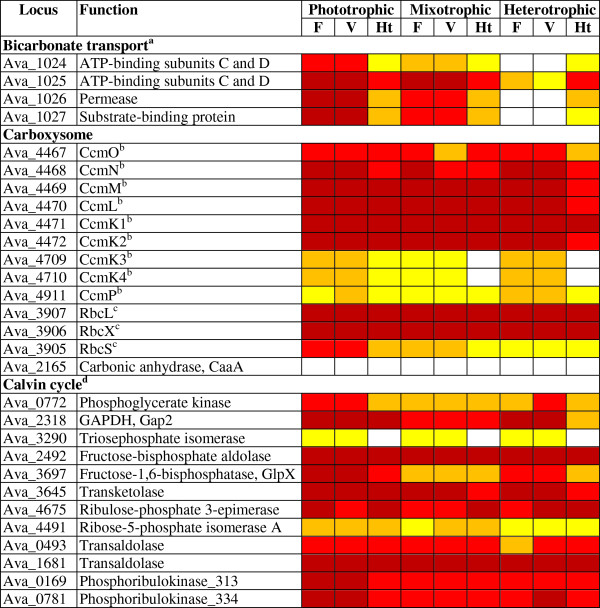
**Transcripts of CO**_**2**_ **-fixation genes.** Colors are as in Figure 5. Legends for superscripts “a” to “d” are in Additional file 8.

In cyanobacteria, ribulose-1,5-bisphosphate carboxylase oxygenase (RuBisCO) is often concentrated in subcompartments—the carboxysomes—in which CO_2_ is concentrated to high levels by a carboxysome-specific carbonic anhydrase [82–84]. Transcript levels of the genes encoding the high-affinity bicarbonate ABC transporter CmpABCD reflect the varying cellular need for CO_2_ in the three growth conditions (Figure 7). Most carboxysome-related transcripts were significantly more abundant in the vegetative cells than in the heterocysts of phototrophic cultures (Figure 7). The abundant transcripts detected for most carboxysomal genes in heterocysts could have been produced in vegetative cells or proheterocysts (immature heterocysts) prior to their differentiation into heterocysts or could have been synthesized in the heterocysts. CcmK1, CcmK2, and CcmM are present at significant levels in the heterocysts of PCC 7120 [26]. The carbonic anhydrase encoded by Ava_2165 is similar to CcaA, the carboxysome-specific carbonic anhydrase in many cyanobacteria, but Ava_2165 was transcribed at background level across experiments. Most Calvin cycle genes were upregulated in the vegetative cells of phototrophic cultures. They were transcribed at significant levels across most experiments, with the exception of Ava_3290, encoding triosephosphate isomerase, whose transcript levels were very low or not distinguishable from background (Figure 7 and Additional file 7).

#### Central carbon metabolism

Most genes encoding glycolysis, pentose phosphate pathway, and tricarboxylic acid cycle enzymes had moderate to high transcript levels across experiments (Additional file 7). Ten of nineteen glycolysis genes, the three known oxidative pentose phosphate pathway genes, and seven of ten genes in the tricarboxylic acid cycle were upregulated 1.5- to 7.4-fold (p ≤ 0.05) in the vegetative cells of phototrophic cultures (Additional file 7). The same genes were not consistently upregulated in the vegetative cells of mixotrophic or heterotrophic cultures. Ava_1682 and Ava_1683 encode, respectively, the oxidative pentose phosphate cycle proteins, glucose-6-phosphate dehydrogenase (G6PD) and OpcA. In *N. punctiforme*, OpcA appears to be an allosteric activator of G6PD and to affect redox modulation of G6PD [85]. The transcript of *zwf* (encoding G6PD) in *N. punctiforme* accumulated 42 h after nitrogen stepdown [86]. In *A. cylindrica* Lemm., the G6PD activity level in heterocysts was estimated to be 67-fold higher than in vegetative cells [87]. Under mixotrophic conditions, transcript levels of Ava_1682 and Ava_1683 were significantly higher in filaments than in vegetative cells and significantly higher in vegetative cells than in heterocysts. These observations suggest that―at least under mixotrophic conditions―transcripts of Ava_1682 and Ava_1683 may be under-represented in heterocysts. Transcript levels of Ava_3044 (encoding 6-phosphofructokinase) were just above background level across cell types in phototrophic and mixotrophic conditions, but were high across cell types in heterotrophic conditions. *A. variabilis* has three glyceraldehyde-3-phosphate dehydrogenases, Gap1, Gap2, and Gap3. The *gap2* transcript was abundant across all experiments, in particular in phototrophic conditions, where it reached 47% of the most abundant transcript in vegetative cells (Figure 7 and Additional file 7, Calvin cycle). This observation is similar to a previous finding that *Synechocystis* PCC 6803 Gap2 is present in multiple growth conditions, with maximum activity in photoautotrophic conditions [88]. Downregulation of *gap2* in heterocysts (Figure 7) also supports a previous report that Gap2 is less abundant in heterocysts than in vegetative cells of *A. variabilis*[89]. However, whereas Valverde et al. [89] detected *gap3* transcripts in heterocysts and vegetative cells of *A. variabilis*, but none of *gap1*, we found the opposite (Additional file 7).

#### Sucrose metabolism

Sucrose is thought to be the principal form of reduced carbon transferred to heterocysts during diazotrophic growth [4, 8, 9, 90–92]. In cyanobacteria sucrose is synthesized by sucrose-phosphate (sucrose-P) synthase and sucrose-P phosphatase [93, 94]. PCC 7120 sucrose-P synthases SpsA and SpsB have different specificities for UDP- and ADP-glucose [93]. In PCC 7120 SpsA is expressed only in vegetative cells and SpsB is expressed in all cells [95]. In our phototrophic cultures, *spsA* was upregulated 6-fold in vegetative cells (p ~ 0.096), in agreement with [95], but it was still transcribed at a low level in heterocysts. In mixotrophic and heterotrophic cultures, *spsA* transcript levels in vegetative cells decreased 31-fold and 14.7-fold, respectively, to a level similar to that in heterocysts (Additional file 7). These observations suggest that *spsA* expression is controlled, at least in part, by the carbon source. In contrast to *spsA*, *spsB* was upregulated 5.6-fold, 1.9-fold, and 35-fold in the heterocysts of phototrophic, mixotrophic, and heterotrophic cultures (p < 0.04), respectively. The sucrose-P phosphatase gene, *sppA* (Ava_2821), was transcribed at low levels across experiments, with a ~ 2-fold upregulation (p ≤ 0.05) in the vegetative cells of phototrophic and mixotrophic cultures (Additional file 7).

Sucrose can be cleaved by invertases, which hydrolyze sucrose irreversibly to fructose and glucose [96], and by sucrose synthases. Sucrose synthases cleave sucrose with UDP in a reversible reaction. Inactivation of the invertase gene, *invB*, whose product is normally strongly expressed in heterocysts, greatly impaired PCC 7120’s growth on N_2_[8, 9]. This result strongly supports the theory that sucrose is the main form of reduced carbon transferred to heterocysts during growth on N_2_. A second alkaline invertase, InvA, which is expressed at a low level in heterocysts, had no such mutant phenotype [8, 9]. *A. variabilis* has a single―neutral―invertase, InvB (Ava_0609). Transcript levels of *invB* were just above background in heterocysts across growth conditions (Additional file 7).

As in related species, *A. variabilis* contains two sucrose synthases, Ava_2283 (SusA) and Ava_3753 (SusB). Curatti et al. [91, 97, 98] suggested that SusA is involved in the conversion of sucrose to polysaccharides in heterocyst-forming cyanobacteria, matching a presumptive function of sucrose synthase in plants [99]. In our experiments, *susA* transcript levels were similar in heterocysts and vegetative cells of phototrophic cultures. While *susA* was upregulated 3.4-fold and 1.9-fold in the vegetative cells of mixotrophic and heterotrophic cultures, respectively, it was still transcribed at 2% to 2.6% of the most highly expressed genes in heterocysts in all conditions. *susA* was also upregulated more than 5-fold in the filaments of mixotrophic cultures compared to phototrophic cultures, as observed by others [91, 97, 98] (Additional file 7). To our surprise, *susB*’s transcript levels reached 18% to 24% of the most highly expressed genes in heterocysts across growth conditions. *susB*’s transcript was 8.1- to 9.6-fold more abundant than that of *susA* in heterocysts of all cultures (Additional file 7), suggesting that *susB* may have different functions in *A. variabilis* and in *Anabaena* sp. PCC 7119. In PCC 7119, a *susB* mutant had no effect on growth or sucrose production [91], whereas a *susA* mutant accumulated more sucrose and less glycogen than the wild-type strain in N_2_-fixing conditions [91, 97, 98].

The *A. variabilis* genome encodes at least two carbohydrate uptake transport (CUT) 1-family ABC transporters, which are specific for disaccharides and oligosaccharides. Among the twelve CUT1-related genes that were identified (Additional file 7), Ava_2748 and Ava_2050 were distinctive. Ava_2748 (membrane protein 2) was upregulated 7- to 31-fold in heterocysts across growth conditions (p ≤ 0.036), and Ava_2050 (ATP-binding protein) was upregulated 7- and 97-fold in heterocysts of phototrophic and heterotrophic cultures, respectively. In all growth conditions, Ava_2050 signal intensities in heterocysts reached at least 26% of that of the most transcribed genes in those cells. These two proteins could be part of an ABC transporter responsible for sucrose uptake in heterocysts. The remaining components of this hypothetical sucrose transporter (i.e., the membrane protein 1 and the substrate-binding protein) cannot be identified with reference only to *A. variabilis* based on our data, because the CUT1-related genes are not clustered in the *A. variabilis* genome. However, the orthologs of Ava_2748 in *N. punctiforme* (Npun_R2792) and *Anabaena* 90 (Ana_C20533) are adjacent to genes Npun_R2793 and Ana_C20534, both of which are orthologs of Ava_0461, annotated as membrane protein 1 in a CUT1 transporter (Additional file 7). Our results show that Ava_0461 is also strongly up-regulated in heterocysts, at least in phototrophic (F, V, Ht: 46 ± 3, 57 ± 15, 187 ± 23 SIU) and heterotrophic conditions (F, V, Ht: 44 ± 6, 49 ± 3; 250 ± 47 SIU). In addition, Ana_C20533 and Ana_C20534 are clustered with Ana_C20535, which is annotated as the periplasmic component of an ABC-type sugar transport system.

#### Glycogen metabolism

The genes for glycogen synthesis enzymes ADP-glucose pyrophosphorylase (Ava_2020), glycogen synthase 1 (Ava_2631), glycogen synthase 2 (Ava_4775), and glycogen branching enzyme (Ava_4616) were upregulated 2.9- to 6.4-fold in the vegetative cells of phototrophic cultures (p ≤ 0.054). The four genes were downregulated in mixotrophic conditions, with transcript levels in filaments 3.6- to 6.3-fold lower in mixotrophic than phototrophic conditions (p ≤ 0.022) (Additional file 7). This last result seems to disagree with an earlier study that showed that mixotrophically-grown filaments contain more glycogen than phototrophically-grown filaments [100]. That early study used 40 mM fructose in the medium, whereas we used only 5 mM fructose. One possible explanation is that a higher supply of fructose and nucleotide sugars improves the kinetics of glycogen synthesis and decreases the need for more enzyme production.

The two genes encoding glycogen phosphorylases (Ava_2996 and Ava_1084) were, as a rule, upregulated in vegetative cells (0.002 ≤ p ≤ 0.06), and slightly upregulated in phototrophic vs. mixotrophic and heterotrophic conditions. In contrast, the debranching enzyme (Ava_2025) gene did not show a cell-specific transcript pattern. The two genes encoding α-phosphoglucomutases (Ava_1737 and Ava_2367) were upregulated over 2.4-fold in the vegetative cells of phototrophic cultures (p ≤ 0.05) and were upregulated up to 3.4-fold in phototrophic vs. mixotrophic and heterotrophic conditions (Additional file 7).

#### Development-related genes

The heterocyst differentiation genes with the highest signal intensities included *devH* (32% to 60% of the most transcribed gene), *sepJ* (9% to 17%), *hetR* (4% to 26%), *hglK* (4% to 17%), *ntcA* (2.8% to 6.1%), *pbpC* (1.6% to 13.5%), *nrrA* (2% to 10%) and *hetN* (1.3% to 8%). Transcript levels for *hetC*, *hetF*, *hetL*, *hetP*, *patA*, *patB*, *patS*, *hepA*, *hepB*, *hepK*, *devC*, *devR*, *pkn30*, and *hgdA* reached at most 2.5% of the most transcribed gene in all experiments (Additional file 7). A clear illustration of the fact that most heterocysts in our steady-state cultures are mature rather than developing heterocysts is the 7- to 25-fold downregulation (p < 0.01) of genes involved in the formation of the heterocyst envelope polysaccharide (*hepA*, Ava_1106, Ava_1108, Ava_1114, Ava_1116, Ava_1120, Ava_1122, and Ava_1124) [13, 101] and of the heterocyst-specific glycolipid layer (*hgdB*, *hgdC*, *hglA*, *hglE*_*A*_, and *hglG*) [13] in the heterocysts of phototrophic cultures (Additional file 7).

#### Genes whose functions have not been characterized experimentally

Many uncharacterized genes were upregulated in heterocysts in at least two growth conditions (Additional file 9). Most of the uncharacterized genes strongly upregulated in heterocysts in phototrophic and heterotrophic conditions did not appear to be upregulated in the heterocysts of mixotrophic cultures. Still, uncharacterized genes that were the most upregulated in heterocysts showed remarkably similar transcript levels in heterocysts across growth conditions (Additional file 9), suggesting that upregulation of these genes in heterocysts is real.

In contrast to the uncharacterized genes upregulated in heterocysts, most uncharacterized genes that were strongly upregulated in vegetative cells were upregulated in a single growth condition (Additional file 9). This observation may be related to our PCA results—growth conditions affected parts of the carbon and energy metabolisms in vegetative cells. Groups of contiguous genes Ava_2383-2385 and Ava_4373-4375 that were transcribed only in vegetative cells and only in phototrophic conditions, and show no similarity to each other, merit note. Those groups encode 80-residue proteins that are 70% to 96% identical to each other, and are found only in a subset of heterocyst-forming cyanobacteria. The transcript level of Ava_2384 reached close to 20% of the most abundant transcript in vegetative cells (Additional file 9).

## Discussion

We report the first comparison of gene expression patterns in the vegetative cells and heterocysts of a filamentous cyanobacterium, *A. variabilis* ATCC 29413. RT-qPCR results showing mean upregulation levels no greater than 14.5-fold for *rbcL* in vegetative cells and no greater than 9.9-fold for *nifK* in heterocysts suggested initially that our RNA extractions were not adequately cell-specific. Cell specificity of our vegetative cell RNA extracts was obscured by the facts that the *rbcL* transcript in heterocysts reached up to 33% of the most transcribed ORF in heterocysts, and that *rbcL* was transcribed at lower levels in vegetative cells under heterotrophic and mixotrophic conditions than under phototrophic conditions. Cell specificity of our heterocyst RNA preparations was obscured by the fact that *nif1* transcripts were apparently targeted for rapid degradation during our isolation of heterocysts. Although substantial transcript levels of *rbcL* in heterocysts are inconsistent with imaging of transcriptional fusions of *rbcL* to luciferase in PCC 7120, which showed at most very slight expression in heterocysts compared to vegetative cells [39], and with diverse other data [4], *A. variabilis* heterocysts might use RuBisCO to reassimilate CO_2_ released by the oxidative pentose phosphate pathway [87, 102].

Still, our microarray results suggest that RNA preparations from heterotrophic cultures were over 90% cell-specific, with eighteen genes upregulated between 10- and 19-fold in vegetative cells and thirty-five genes upregulated between 30- and 167-fold in heterocysts. Similarly, transcript levels of Ava_4669 and Ava_2050 were over 200-fold higher in the heterocysts than in the filaments of phototrophic cultures (Additional file 9). At least one gene, Ava_2687 (encoding an arsenate reductase-related protein), was strongly upregulated in heterocysts across growth conditions (60-, 24-, and 58-fold in phototrophic, mixotrophic, and heterotrophic cultures, respectively). This gene could be used as an alternative probe to *nifK* to determine the cell specificity of heterocyst RNA extractions. In contrast, we found no gene that would be a consistently strong, specific probe for vegetative cell RNA extracts across the three tested growth conditions. Because genes highly upregulated in vegetative cells were in no instance highly upregulated in all three growth conditions, a more reliable approach for future studies will likely be to use different RT-qPCR probes for vegetative cells for different conditions, e.g., Ava_2383 for phototrophic conditions, Ava_2928 for mixotrophic conditions, and Ava_3729 for heterotrophic conditions.

### Development-related genes and certain uncharacterized genes

Our analysis was not expected to illuminate the process of heterocyst differentiation, per se, for three interrelated reasons: (i) Steady state cultures were needed for prospective metabolic flux analysis. (ii) RNA extracted from vegetative cells in steady state cultures is combined with RNA extracted from proheterocysts that have not yet formed the envelope that protects against cavitation. Presumably as a result, genes involved in early, transient developmental processes such as deposition of the heterocyst envelope appear upregulated in vegetative cells (Additional file 7). (iii) Correspondingly, RNA is isolated from mature or almost fully mature heterocysts only when their envelope protects them extensively against cavitation. Therefore, our analysis provides the opportunity to highlight ongoing processes characteristic of mature heterocysts, including their interactions with vegetative cells, and to gain further insight into the physiological differences between mature heterocysts and vegetative cells. To those ends, we have provided (in Additional file 7) a section on known heterocyst developmental genes and (in Additional file 9) sections on differentially transcribed genes. Among these genes are Ava_2748 and Ava_2050, which are likely parts of one or two carbohydrate uptake transport (CUT) 1-family ABC transporters, known to be specific for di- and oligosaccharides. These two genes represent possible members of the yet unknown sucrose transporter. Although we recognize that under phototrophic conditions, transcript levels in heterocysts are inflated because of the low *nif1* transcript levels, the 204-fold and 35-fold higher transcript levels for Ava_2050 and Ava_2748, respectively, in heterocysts than in filaments still suggest that these genes are upregulated in heterocysts in these conditions. Other genes from Additional file 9 that have high transcript levels, and are strongly upregulated, in heterocysts may also warrant inquiry.

Under phototrophic conditions, 77% of the genes with unknown products that were upregulated in one type of cell versus the other were upregulated in heterocysts. Despite the limitations of our data (e.g., degradation of the *nif1* transcripts), these observations suggest to us that much of what is unknown about the metabolism of phototrophic, N_2_-fixing cultures takes place in heterocysts.

### Amino acid biosynthesis

The current model for nitrogen assimilation after N_2_ fixation has NH_3_ incorporated into Gln by glutamine synthetase (encoded by *glnA*) in heterocysts [6, 17]. Gln is then transported to vegetative cells, where—together with 2-oxoglutarate—it serves as substrate for glutamate synthase (encoded by *glsF*) to produce two Glu [6, 7, 103]. Finally, one Glu is transported back to the heterocysts, where it becomes the substrate of glutamine synthetase for Gln synthesis. The Gln-Glu exchange between vegetative cells and heterocysts results in a net nitrogen flux to the vegetative cells. The second glutamate synthase substrate, 2-oxoglutarate, may also be produced in heterocysts by isocitrate dehydrogenase [7, 104]. In PCC 7120, glutamine synthetase activity is regulated by protein-protein interactions with the inhibitory protein IF7 (encoded by *gifA*), whose expression is negatively controlled by NtcA [105]. In *A. variabilis*, IF7 is encoded by Ava_0148, adjacent to *glnA*. Glutamate dehydrogenase (encoded by *gdhA*) does not seem to play an important role in nitrogen assimilation [103, 106], but it can give a competitive advantage in non-exponential phases of growth, possibly related to the fact that ATP is not required for its activity [107]. Other authors [108] suggest that Arg may be an alternative carrier of fixed nitrogen from heterocysts.

Prior results [6, 39] with *A. cylindrica* and PCC 7120 led to the expectation that under photoautotrophic conditions, glutamine synthetase would be active in both types of cells whereas glutamate synthase would be active specifically in vegetative cells. However, in *A. variabilis*, glutamine synthetase, glutamate synthase, and isocitrate dehydrogenase appear to be actively transcribed in both types of cells. Our results therefore suggest that Glu and Gln, as well as Ser, Gly, Cys, Thr, and Pro, are actively produced in heterocysts. For all other protein amino acids, our results are inconclusive. The phenotypes of several amino acid ABC transporter mutants of PCC 7120 are known [65–68], but molecules as large as 623-Da calcein may move back and forth passively between vegetative cells and heterocysts, perhaps through the septosome, without transiting through the periplasm [69, 70]. Until it has been proven or disproven that amino acids are transported through the septosome, the phenotypes of amino acid ABC transporters cannot be used to help reach a conclusion that a particular amino acid is or is not synthesized in heterocysts.

It is not excluded that only certain steps of amino acid synthesis take place in heterocysts, with precursors being provided by vegetative cells. Because heterocysts funnel much of their energy and redox power into N_2_ fixation, some redox or energy-intensive enzymatic steps might take place only in vegetative cells. For example, the two genes in the Lys pathway that are transcribed at the lowest levels in heterocysts encode enzymes that require ATP (aspartate kinase) and NAD(P)H (dihydrodipicolinate reductase) (Figure 5). Future metabolic flux analyses with diazotrophically grown *A. variabilis* must test for the possible transport of Ala, Asn, Asp, Lys, Arg, Phe, Tyr, Trp, Ile, Val, Leu, His, and Met (or their precursors) between cell types.

#### Photosynthesis

In agreement with our results, peptides corresponding to PsbO, PsbU, and PsbV were detected in heterocysts of PCC 7120 at frequencies above 50% of those observed in vegetative cells (Table S4 in [26]). Even though PS II likely does not evolve O_2_ in heterocysts, our results support the hypothesis that complete or incomplete PS II complexes have an as yet unknown function in heterocysts. A cyclic electron flow could exist in heterocyst PS II [109], serving as a further source of ATP, and [4] an electron carrier (e.g., sulfide or ferrous ions) might shuttle electrons between vegetative cells and heterocyst PS II.

### CO_2_ fixation

*A. variabilis* CcmM has an N-terminal carbonic anhydrase domain followed by three RbcS-like domains. In *Thermosynechococcus elongatus*, which lacks a *ccaA* homolog, CcmM is the carboxysomal carbonic anhydrase [84]. PCC 7120 and *A. variabilis* CcmM proteins contain all of the conserved residues thought to be required for *T. elongatus* CcmM carbonic anhydrase activity [84]. Because PCC 7120 lacks a *ccaA* homolog and because *ccaA* is barely transcribed in *A. variabilis*, CcmM―with high to very high transcript levels in vegetative cells―may be the carboxysome-specific carbonic anhydrase in PCC 7120 and *A. variabilis*. In *Synechococcus* PCC 7942, the average RuBisCO complex contains eight RbcL molecules and five RbcS molecules, and CcmM has been shown to form complexes readily with RbcL [110]. It is tempting to speculate that in vegetative cells of *A. variabilis*, CcmM assembles with incomplete RbcL-RbcS complexes to form a bi-functional RuBisCO-carbonic anhydrase. Such an assembly might permit efficient channeling of CO_2_ from carbonic anhydrase to RuBisCO. It would also explain why the *rbcS* transcript was 13-fold less abundant than the *rbcL* transcript in the vegetative cells of phototrophic cultures (Figure 7).

## Conclusions

Derived from RNAs purified after lengthy cell separation procedures, our microarray data cannot compare in quality and applicability with others generated with rapidly extracted, more intact RNAs. Despite this limitation, our results show that the carbon source or sources significantly affect transcription patterns in vegetative cells but not as much in heterocysts. This finding agrees with the respective functions of vegetative cells and heterocysts. Our results help to clarify which amino acids are actively produced in heterocysts, and which may require transport from cell to cell. We hope to use this information to build a filament-level metabolic network model for metabolic flux analyses. This first direct comparison of transcript levels in heterocysts and vegetative cells also allowed us to identify many uncharacterized genes that are differentially regulated in the two cell types and that warrant further characterization.

## Availability of supporting data

The data set supporting the results of this article is available in the National Center for Biotechnology Information Gene Expression Omnibus database, at http://www.ncbi.nlm.nih.gov/geo/ under accession number GSE46076.

## Electronic supplementary material

Additional file 1: **Gene-specific primers used for RT-qPCR.** This table lists the primers used for RT-qPCR to test the cell specificity of RNA extractions. The *rnpB* gene was used as an internal control for data normalization. *rbcL* and *nifK* were used as a vegetative cell-specific gene and a heterocyst-specific gene, respectively. F: forward; R: reverse. (PDF 38 KB)

Additional file 2: Figure S1: Bioanalyzer analysis of RNA quality in RNA samples extracted from phototrophic (P), mixotrophic (M), and heterotrophic (H) cultures. The RNA samples prepared from filaments, vegetative cells, and heterocysts from the three growth conditions were analyzed on a Bioanalyzer prior to being used for microarray experiments. **Figure S2.** Volcano plots of ratios of transcript levels in heterocysts divided by corresponding levels in vegetative cells in phototrophic (P), mixotrophic (M), and heterotrophic (H) cultures. The variation in transcript levels is expressed as the log_2_ of the ratio of transcripts in heterocysts divided by transcripts in vegetative cells, and is plotted versus the statistical significance of the variation, expressed as log_10_ of the *p*-value. Two-fold expression changes and *p*-values of 0.01 are indicated by red lines. (PDF 458 KB)

Additional file 3: **Normalized microarray data.** This table lists the normalized microarray data for our twenty seven experiments, representing the three biological replicates of experiments performed with RNA extracted from whole filaments, vegetative cells, and isolated heterocysts from phototrophic cultures, as well as from mixotrophic and heterotrophic cultures. Each gene is identified by its microarray sequence ID, its locus number, its predicted function, and its functional category. Fourteen categories were based on the Kyoto Encyclopedia of Genes and Genomes (KEGG) pathway database [48], Blastp searches, and previous publications of gene functions. ORFs annotated only with a protein domain name were arbitrarily included in the Other functions category and those annotated as hypothetical proteins or proteins of unknown function were arbitrarily grouped in the Unknown category. (XLSX 2 MB)

Additional file 4: **R** ^**2**^ **values between all pairs of microarray experiments.** This table lists the coefficients of determination (R^2^ values) between the twenty seven experiments. R^2^ values show that reproducibility was high for biological replicates of the same RNA extractions. In boldface and highlighted in green: R^2^ values between biological replicates of identical RNA extractions. Highlighted in yellow: R^2^ values between heterocyst RNAs extracted from different culture conditions. BR: biological replicate. (PDF 67 KB)

Additional file 5: **Reduced data and weighted residuals for phototrophic, mixotrophic, and heterotrophic conditions.** Reduced data are the normalized data once genes described in Additional file 6 have been deleted. The reduced data sets contain 3,949 genes in phototrophic conditions, 3,885 genes in mixotrophic conditions, and 3,933 genes in heterotrophic conditions. The weighted residuals were calculated using equation 1 with the a and b values determined by linear regression (Additional file 6). (XLSX 4 MB)

Additional file 6: **Data reduction and linear modeling.** Additional file 6 describes how data for genes that are not expressed and genes that show inconsistent transcript levels were removed from the normalized data. Additional file 6 also details the results of linear modeling of the transcript data in each growth condition as applied to the reduced data using the function F_i_ = aV_i_ + bHt_i_ - 1, where F_i_, V_i_, and Ht_i_ represent the means of gene i transcript levels in filaments, vegetative cells, and heterocysts, respectively; a and b are constants that reflect the relative abundance of vegetative cells and heterocysts in the filaments; and -1 is a term that forces the intercept to 0. (PDF 29 KB)

Additional file 7: **Genes discussed in this study.** This table lists all genes discussed in the manuscript. Each gene is identified by its locus number, and its predicted function. Microarray data are reported as averages of the three biological replicates for each experiment. (XLSX 253 KB)

Additional file 8: **Supplementary legend for Figure** 5 **.** Supplementary legend for Figure 7. (PDF 70 KB)

Additional file 9: **Top uncharacterized genes upregulated in heterocysts and in vegetative cells.** This spreadsheet lists the uncharacterized genes with the highest upregulation in heterocysts and in vegetative cells in each growth condition, based on the ratio of transcript levels between vegetative cells and heterocysts. Each gene is represented by its locus number and its predicted function. (XLSX 20 KB)
