# Supplementary material for: Cell-specific gene expression in Anabaena variabilis grown phototrophically, mixotrophically, and heterotrophically
Source: BMC Genomics. 2013 Nov 5;14(1):759. doi: 10.1186/1471-2164-14-759 (PMC4046671; doi:10.1186/1471-2164-14-759)
Supplement: Supplementary file 1 — Additional file 1: Gene-specific primers used for RT-qPCR. This table lists the primers used for RT-qPCR to test the cell specificity of RNA extractions. The rnpB gene was used as an internal control for data normalization. rbcL and nifK were used as a vegetative cell-specific gene and a heterocyst-specific gene, respectively. F: forward; R: reverse. (PDF 38 KB) [file 12864_2013_5475_MOESM1_ESM.pdf]

Additional file 1 – Table S1

Table S1. Gene-specific primers used for RT-qPCR; F: forward; R: reverse.

Primer name	Sequence
*rnpB*_F	5'- GCG AGC GAT CGT GAG GAT A -3'
*rnpB*_R	5'- GCA CCT TTG CAC CCT TAC CA -3'
*rbcL*_F	5'- CGC GGT GGT TTG GAC TTC -3'
*rbcL*_R	5'- CGC GCC ATC TTT GGA ATG -3'
*nifK*_F	5'- CGC GAT ACC AAG ATC CCA AT -3'
*nifK*_R	5'- CCG AGA GTA GAA TAA CGG TGT AAG TG -3'
