# Supplementary material for: Cell-specific gene expression in Anabaena variabilis grown phototrophically, mixotrophically, and heterotrophically
Source: BMC Genomics. 2013 Nov 5;14(1):759. doi: 10.1186/1471-2164-14-759 (PMC4046671; doi:10.1186/1471-2164-14-759)

Additional file 2 – Figures S1 and S2

Figure S1 Bioanalyzer analysis of RNA quality in RNA samples extracted from phototrophic (P), mixotrophic (M), and heterotrophic (H) cultures
Ma: RNA molecular markers in nt; F1−F3, V1−V3, and Ht1−Ht3: biological replicates of RNA extracted from whole filaments, vegetative cells, and heterocysts, respectively. Panels P_Ht1, P_Ht2, and P_Ht3: Electrophoretograms of the RNA samples isolated from the heterocysts of phototrophic cultures. These profiles correspond to samples Ht1, Ht2, and Ht3 in panel P. RNA ladder: Electrophoretogram of the Agilent RNA size markers. Marker sizes are in nt. FU: arbitrary fluorescence units for RNA concentration.

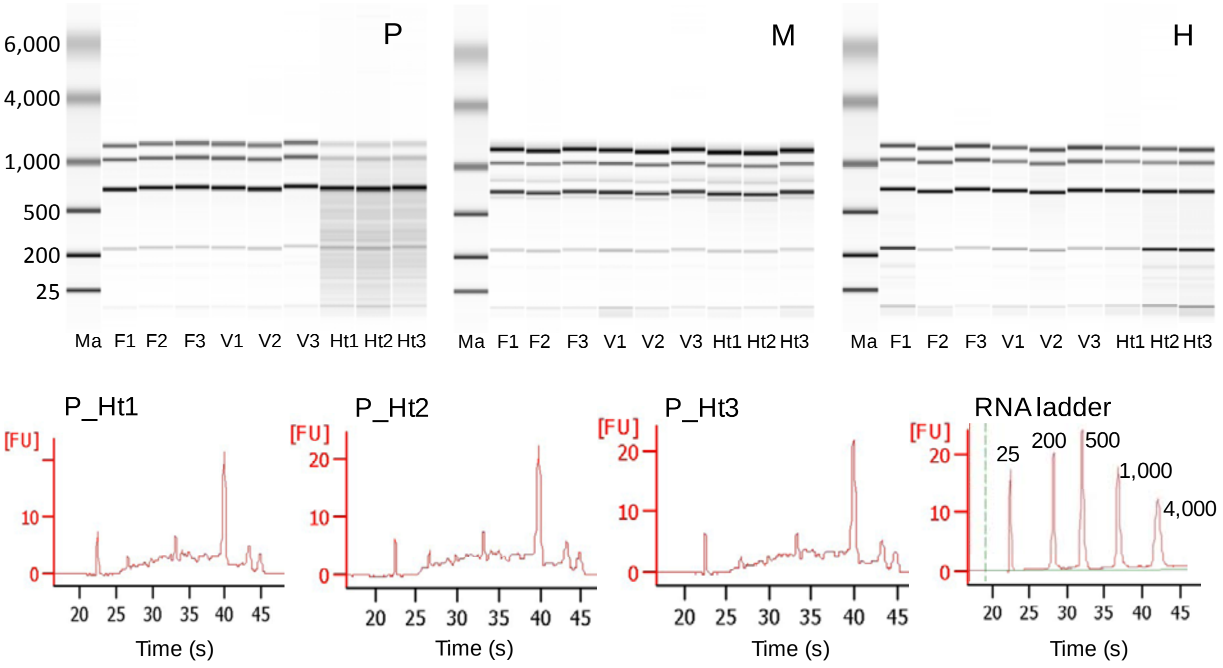

Additional file 2 – Figures S1 and S2

Figure S2 Volcano plots of ratios of transcript levels in heterocysts divided by corresponding levels in vegetative cells in phototrophic (P), mixotrophic (M), and heterotrophic (H) cultures
The variation in transcript levels is expressed as the $\log_2$ of the ratio of transcripts in heterocysts divided by transcripts in vegetative cells, and is plotted versus the statistical significance of the variation, expressed as $\log_{10}$ of the *p*-value. Two-fold expression changes and *p*-values of 0.01 are indicated by red lines.

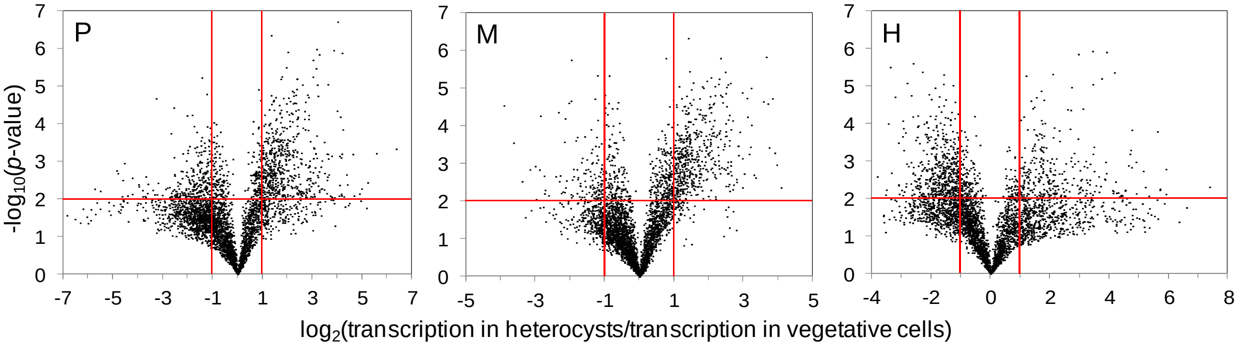

Supplement: Supplementary file 2 — Additional file 2: Figure S1: Bioanalyzer analysis of RNA quality in RNA samples extracted from phototrophic (P), mixotrophic (M), and heterotrophic (H) cultures. The RNA samples prepared from filaments, vegetative cells, and heterocysts from the three growth conditions were analyzed on a Bioanalyzer prior to being used for microarray experiments. Figure S2. Volcano plots of ratios of transcript levels in heterocysts divided by corresponding levels in vegetative cells in phototrophic (P), mixotrophic (M), and heterotrophic (H) cultures. The variation in transcript levels is expressed as the log2 of the ratio of transcripts in heterocysts divided by transcripts in vegetative cells, and is plotted versus the statistical significance of the variation, expressed as log10 of the p-value. Two-fold expression changes and p-values of 0.01 are indicated by red lines. (PDF 458 KB) [file 12864_2013_5475_MOESM2_ESM.pdf]
