# Supplementary material for: Cell-specific gene expression in Anabaena variabilis grown phototrophically, mixotrophically, and heterotrophically
Source: BMC Genomics. 2013 Nov 5;14(1):759. doi: 10.1186/1471-2164-14-759 (PMC4046671; doi:10.1186/1471-2164-14-759)
Supplement: Supplementary file 4 — Additional file 4: R 2 values between all pairs of microarray experiments. This table lists the coefficients of determination (R2 values) between the twenty seven experiments. R2 values show that reproducibility was high for biological replicates of the same RNA extractions. In boldface and highlighted in green: R2 values between biological replicates of identical RNA extractions. Highlighted in yellow: R2 values between heterocyst RNAs extracted from different culture conditions. BR: biological replicate. (PDF 67 KB) [file 12864_2013_5475_MOESM4_ESM.pdf]

Additional file 4 – R^2 values between all pairs of microarray experiments

R^2 values between all pairs of microarray experiments. Boldface and highlighted in green: R^2 values between biological replicates of identical RNA extractions. Highlighted in yellow: R^2 values between heterocyst RNAs extracted from different growth conditions. BR: biological replicate.

Sample		P_F			P_V			P_Ht			M_F			M_V			M_Ht			H_F			H_V			H_Ht		
	BR	1	2	3	1	2	3	1	2	3	1	2	3	1	2	3	1	2	3	1	2	3	1	2	3	1	2	3
P_F	1	**1**	**0.998**	**0.984**	0.987	0.991	0.877	0.533	0.549	0.508	0.539	0.532	0.571	0.556	0.592	0.605	0.424	0.436	0.411	0.635	0.681	0.674	0.679	0.666	0.683	0.459	0.435	0.430
	2		**1**	**0.983**	0.986	0.992	0.875	0.536	0.552	0.511	0.539	0.531	0.570	0.557	0.593	0.605	0.427	0.438	0.413	0.637	0.684	0.678	0.680	0.668	0.686	0.462	0.438	0.433
	3			**1**	0.993	0.964	0.884	0.530	0.546	0.499	0.538	0.546	0.573	0.545	0.595	0.601	0.413	0.426	0.406	0.624	0.662	0.650	0.661	0.643	0.663	0.447	0.418	0.428
P_V	1				**1**	**0.969**	**0.883**	0.529	0.542	0.494	0.533	0.538	0.567	0.541	0.587	0.597	0.409	0.421	0.400	0.628	0.667	0.656	0.666	0.645	0.667	0.446	0.420	0.424
	2					**1**	**0.857**	0.533	0.549	0.514	0.535	0.521	0.564	0.561	0.586	0.600	0.430	0.441	0.414	0.637	0.684	0.680	0.679	0.676	0.688	0.464	0.444	0.432
	3						**1**	0.715	0.730	0.681	0.797	0.797	0.823	0.762	0.802	0.816	0.615	0.621	0.612	0.654	0.672	0.656	0.690	0.698	0.695	0.637	0.584	0.624
P_Ht	1							**1**	**0.994**	**0.984**	0.808	0.785	0.801	0.887	0.880	0.875	0.961	0.968	0.953	0.585	0.576	0.569	0.588	0.627	0.607	0.964	0.862	0.941
	2								**1**	**0.986**	0.818	0.792	0.810	0.891	0.887	0.883	0.962	0.969	0.957	0.588	0.583	0.575	0.595	0.635	0.615	0.960	0.857	0.939
	3									**1**	0.801	0.772	0.791	0.893	0.863	0.855	0.969	0.974	0.957	0.575	0.556	0.551	0.570	0.621	0.594	0.969	0.877	0.946
M_F	1										**1**	**0.970**	**0.992**	0.920	0.907	0.923	0.799	0.792	0.801	0.602	0.594	0.577	0.619	0.678	0.637	0.798	0.733	0.797
	2											**1**	**0.979**	0.895	0.889	0.890	0.760	0.754	0.769	0.611	0.575	0.553	0.615	0.666	0.624	0.764	0.713	0.786
	3												**1**	0.912	0.908	0.921	0.781	0.776	0.783	0.627	0.618	0.600	0.643	0.697	0.661	0.785	0.723	0.785
M_V	1													**1**	**0.955**	**0.962**	0.86	0.871	0.860	0.610	0.591	0.583	0.616	0.669	0.635	0.871	0.797	0.854
	2														**1**	**0.975**	0.84	0.852	0.850	0.601	0.609	0.596	0.617	0.655	0.638	0.840	0.735	0.825
	3															**1**	0.843	0.852	0.848	0.600	0.617	0.608	0.629	0.665	0.646	0.839	0.735	0.814
M_Ht	1																**1**	**0.995**	**0.988**	0.514	0.509	0.505	0.522	0.577	0.544	0.973	0.860	0.944
	2																	**1**	**0.987**	0.521	0.517	0.513	0.528	0.578	0.551	0.976	0.860	0.945
	3																		**1**	0.488	0.483	0.475	0.497	0.550	0.518	0.953	0.827	0.938
H_F	1																			**1**	**0.942**	**0.930**	0.967	0.947	0.964	0.597	0.671	0.602
	2																				**1**	**0.994**	0.971	0.933	0.984	0.580	0.602	0.551
	3																					**1**	0.960	0.922	0.977	0.575	0.596	0.539
H_V	1																						**1**	**0.962**	**0.985**	0.597	0.646	0.584
	2																							**1**	**0.973**	0.649	0.700	0.637
	3																								**1**	0.619	0.655	0.598
H_Ht	1																									**1**	**0.936**	**0.975**
	2																										**1**	**0.945**
	3																											**1**
