# Supplementary material for: Cell-specific gene expression in Anabaena variabilis grown phototrophically, mixotrophically, and heterotrophically
Source: BMC Genomics. 2013 Nov 5;14(1):759. doi: 10.1186/1471-2164-14-759 (PMC4046671; doi:10.1186/1471-2164-14-759)
Supplement: Supplementary file 6 — Additional file 6: Data reduction and linear modeling. Additional file 6 describes how data for genes that are not expressed and genes that show inconsistent transcript levels were removed from the normalized data. Additional file 6 also details the results of linear modeling of the transcript data in each growth condition as applied to the reduced data using the function Fi = aVi + bHti - 1, where Fi, Vi, and Hti represent the means of gene i transcript levels in filaments, vegetative cells, and heterocysts, respectively; a and b are constants that reflect the relative abundance of vegetative cells and heterocysts in the filaments; and -1 is a term that forces the intercept to 0. (PDF 29 KB) [file 12864_2013_5475_MOESM6_ESM.pdf]

Additional file 6: Data reduction and linear modeling

In N_2-fixing *A. variabilis* filaments, transcript levels of any gene, i, should verify the equation, $F_i = aV_i + bHt_i$. Assuming that heterocysts and vegetative cells contain similar amounts of RNA, and assuming that RNA is extracted with the same yield from whole filaments, vegetative cells, and heterocysts, a + b should equal 1, with a between 0.9 and 1, and b between 0 and 0.1. Statistical methods were used to determine the values of a and b that best fit our data for each of the three growth conditions.

Prior to linear modeling (and generation of volcano plots), the sets of transcript data for the three growth conditions were reduced by removing three types of genes. First, on the basis of the distribution of signal intensities (Fig. 2, left panels), all genes that showed average transcript levels below 128 ($\log_2 128 = 7$) across all cell types and growth conditions (1,182 genes, total) were removed to yield a set of 4,474 expressed genes. The remaining data reduction steps were performed independently for each growth condition, generating different sets of reduced data for each of the three growth conditions. Second, genes with standard deviations (SD) above 50% of the average signal intensity in at least one of the data for filaments, vegetative cells, and heterocysts were removed. Third, genes were also removed whose V_i exhibited high SD (i.e., [SD of V_i]/[mean of V_i] > 0.3) and whose V_i/F_i ratios were > 1.2 or < 0.83. The final reduced data sets contained 3,949 genes in phototrophic conditions, 3,885 genes in mixotrophic conditions, and 3,933 genes in heterotrophic conditions. The distributions of transcript levels in these data sets are shown in Fig. 2 (right panels).

For phototrophic conditions, linear modeling of the reduced data set to the equation $F_i = aV_i + bHt_i -1$ (where the term -1 constrains the intercept to 0), gave a = 1.023 ± 0.005 and b = 0.022 ± 0.006, with a multiple R^2 value of 0.975. This value of the coefficient of multiple determination R^2 close to 1 [1] suggests that $F_i = 1.023V_i + 0.022Ht_i$ is an excellent model for

our data. Linear modeling applied to the reduced data set for mixotrophic conditions gave a = 0.930 ± 0.011 and b = 0.057 ± 0.011, with a multiple R^2 value of 0.942. Again, this R^2 value suggests that $F_i = 0.93V_i + 0.057Ht_i$ is an excellent model for our data. For heterotrophic conditions, linear modeling of the reduced data set gave a = 1.035 ± 0.004 and b = -0.053 ± 0.005.

The majority of *A. variabilis* genes have similar transcript levels in vegetative cells and heterocysts. Spearman's rank correlation coefficient is a non-parametric measure of statistical dependence between two variables [1]. Values close to 1 indicate a high correlation level between data, whereas a value close to 0 indicates absence of correlation. The Spearman's rank correlation coefficients calculated between transcript data from vegetative cells and heterocysts (0.73, 0.90, and 0.74 for phototrophic, mixotrophic, and heterotrophic conditions, respectively), confirm that transcript levels in vegetative cells and heterocysts are highly correlated. These high degrees of correlation (also called multi-collinearity) likely affect the calculation of a and b by masking the positive contribution of transcript levels in heterocysts to transcript levels in filaments, and may explain the negative b value obtained for heterotrophic conditions. Grid searching was used as a second approach to model the transcript results for heterotrophic conditions to $F_i = aV_i + bHt_i$. Grid searching used 100 different values of a and b. To force $b > 0$, a and b values were constrained in the ranges $0.8 < a < 1.2$ and $0.0000 < b < 0.1$. Bootstrapping (1,000 rounds, sample size of 565 genes) was used to calculate confidence intervals for a and b. Bootstrapping is a resampling method that estimates the precision of sample values [2]. Values of a and b obtained after grid searching and bootstrapping were a = 1.071 ± 0.013 and b = 0.00011 ± 0.00078. In contrast to the a and b parameters obtained for phototrophic and mixotrophic conditions, which have SD values between 0.5% and 27% of the mean, the SD for b (seven times the mean) in heterotrophic conditions suggests that this b value is not reliable.

REFERENCES

1. Sokal RR, RohlF FJ: *Biometry. The Principles and Practice of Statistics in Biological Research*, 2nd edn. New York: W. H. Freeman and Company; 1981.

2. Efron B, Tibshirani RJ: *An Introduction to the Bootstrap*. Boca Raton: Chapman & Hall; 1993.
