# Supplementary material for: Cell-specific gene expression in Anabaena variabilis grown phototrophically, mixotrophically, and heterotrophically
Source: BMC Genomics. 2013 Nov 5;14(1):759. doi: 10.1186/1471-2164-14-759 (PMC4046671; doi:10.1186/1471-2164-14-759)
Supplement: Supplementary file 8 — Additional file 8: Supplementary legend for Figure 5 . Supplementary legend for Figure 7. (PDF 70 KB) [file 12864_2013_5475_MOESM8_ESM.pdf]

Additional file 8

Supplementary legend for Figure 5

[a] *glnA* was down-regulated 2.3-fold in the heterocysts of phototrophic cultures ($p = 0.04$). [b] *gifA's* transcript reached about 71% of the most abundant transcript in heterocysts across growth conditions. It was upregulated over 12-fold in the heterocysts of phototrophic cultures. [c] *glsF* was never significantly upregulated in vegetative cells. [d] *gdhA* was upregulated 4.2-fold in the heterocysts of phototrophic cultures ($p < 0.01$). [e] Radioactive Ala is already detectable after 1 s of $^{13}NH_4^+$ assimilation in *A. variabilis*, but its accumulation is strongly inhibited by L-methionine-DL-sulfoximine, suggesting that Ala is produced by transamination [1]. Any of these three aminotransferases could be involved in Ala or Asp synthesis. [f] The gene encoding phosphoserine phosphatase is unknown, but phosphoserine phosphatase activity in crude extracts of heterocysts of phototrophic cultures was 41.3 ± 13.3 nmol min^{-1} mg^{-1} protein versus 21.7 ± 0.1 nmol min^{-1} mg^{-1} protein in extracts of whole filaments. [g] Glycine is produced from Ser in one step. [h] One serine O-acetyltransferase gene (out of three) and one cysteine synthase gene (out of four) were transcribed across experiments, in agreement with the fact that isolated heterocysts can synthesize Cys from sulfide [2]. [i] All other genes in the Lys pathway were transcribed across cell types and growth conditions. In cyanobacteria, Lys synthesis takes place through the aminotransferase pathway [3]. [j] All other genes in the Arg pathway had transcript levels equal to or greater than those of Ava_3118. [k] Two of the four 3-deoxy-7-phosphoheptulonate synthase genes were transcribed across experiments. With the exception of chorismate synthase, all other genes for chorismate synthesis were transcribed across experiments. [l] Downstream of chorismate, phenylalanine is synthesized through phenylpyruvate and tyrosine synthesis is synthesized through arogenate [4]; however, the aminotransferase responsible for arogenate synthesis is unknown. One of the two histidinol-phosphate aminotransferase genes was transcribed across

experiments. [m] Genes encoding anthranilate synthase components and Trp synthase subunits were transcribed in all experiments, but the N-(5'-phosphoribosyl)anthranilate isomerase gene had background level transcripts across experiments. [n] The three genes in the Pro pathway have transcripts that accumulated at very low to moderate levels across experiments. [o] All genes in the Thr pathway (see additional file 8) were transcribed across experiments. [p] A catabolic–type threonine dehydratase gene was transcribed in conditions where the anabolic threonine dehydratase gene was not. The three other known genes in the Val and Ile pathways were transcribed across experiments. [q] The three other known genes in the Leu pathway from 2-oxovalerate were transcribed at high levels across experiments. [r] The gene encoding the *A. variabilis* histidinol-phosphate phosphatase is unknown. Of the nine other genes in the His pathway (Additional file 8) Ava_3285 was the only one with background transcript levels across experiments. Mutating one of the two PCC 7120 *hisD* genes results in a Fox^- phenotype [5], suggesting that His biosynthesis plays an important role in heterocyst structure and/or function. [s] Isolated heterocysts of *A. variabilis* cannot produce Met from $^{35}S^{2-}$ or $[^{35}S]$Cys, suggesting that heterocysts import their Met from vegetative cells [2]. Methionine synthase could participate in S-adenosylmethionine (AdoMet) synthesis in heterocysts. (The genes involved in AdoMet synthesis and L-homo-Cys regeneration from AdoMet were all transcribed in heterocysts.)

Supplementary legend for Figure 7

[a] In phototrophic cultures *cmpABCD* were upregulated over 10-fold in vegetative cells compared to heterocysts ($p < 0.01$). Transcript levels in vegetative cells decreased only 5- to 10-fold ($p < 0.01$) in mixotrophic cultures, but 33- to 350-fold ($p < 0.01$) in heterotrophic cultures, suggesting that mixotrophic cultures still partially rely on CO_2 fixation for growth, but that heterotrophic

cultures rely entirely on fructose for carbon and energy. [b] *ccmK1*,*M*, *L*, *K2*, *N*, and *O* were upregulated 2- to 3.5-fold in vegetative cells ($p < 0.01$). *ccmK3*, *K4*, and *P* were upregulated in vegetative cells, but with lower confidence intervals ($p < 0.06$). In mixotrophic conditions, carboxysome-related transcripts were less abundant than in phototrophic conditions, with transcript abundance 2- to 3-fold lower than in phototrophic conditions. Surprisingly, most carboxysomal genes were transcribed in heterotrophic cultures at levels intermediate between the phototrophic and mixotrophic cultures. Transcripts of carboxysomal genes were still abundant in heterocysts. The *ccmK1*, *K2*, *L*, and *M* transcripts reached 11% to 24% of the most abundant heterocyst transcripts in phototrophic cultures. The same holds true for heterocysts of mixotrophic and heterotrophic cultures, although with slightly lower transcription levels. [c] *rbcL* was upregulated in the vegetative cells of phototrophic (2.7-fold, $p < 0.001$) and mixotrophic (2.4-fold, $p < 0.003$) cultures. The *rbcL* transcript still reached 22% to 30% of the most abundant transcript in heterocysts across growth conditions. Despite *rbcLXS* being likely cotranscribed, the *rbcS* transcript was 13-fold less abundant than the *rbcL* transcript in the vegetative cells of phototrophic cultures. [d] Most Calvin cycle genes were upregulated in the vegetative cells of phototrophic cultures, with the genes encoding phosphoglycerate kinase ($p < 0.02$), Calvin cycle-specific Gap2 ($p < 0.01$) [6], triosephosphate isomerase ($p < 0.001$), fructose bisphosphate aldolase ($p < 0.003$) [7], transketolase ($p < 0.003$), and both phosphoribulokinases ($p < 0.001$ and < 0.05) being upregulated between 2- and 3-fold. Transcription of most of these genes decreased in mixotrophic and heterotrophic cultures.

Additional file 8

REFERENCES

1. Meeks JC, Wolk CP, Lockau W, Schilling N, Shaffer PW, Chien WS: **Pathways of assimilation of ^{13}N-N_2 and $^{13}NH_4^+$ by cyanobacteria with and without heterocysts**. *J Bacteriol* 1978, **134**:125−130.
2. Giddings TH, Jr, Wolk CP, Shomer-Ilan A: **Metabolism of sulfur compounds by whole filaments and heterocysts of *Anabaena variabilis***. *J Bacteriol* 1981, **146**:1067−1074.
3. Hudson AO, Singh BK, Leustek T, Gilvarg C: **An LL-diaminopimelate aminotransferase defines a novel variant of the lysine biosynthesis pathway in plants**. *Plant Physiol* 2006, **140**:292−301.
4. Hall GC, Flick MB, Gherna RL, Jensen RA: **Biochemical diversity for biosynthesis of aromatic amino acids among the cyanobacteria**. *J Bacteriol* 1982, **149**:65−78.
5. Lechno-Yossef S, Fan Q, Wojciuch E, Wolk CP: **Identification of ten *Anabaena* sp. genes that under aerobic conditions are required for growth on dinitrogen but not for growth on fixed nitrogen**. *J Bacteriol* 2011, **193**:3482−3489.
6. Valverde F, Peleato ML, Fillat MF, Gomez-Moreno C, Losada R, Serrano A: **Simultaneous occurrence of two different glyceraldehyde-3-phosphate dehydrogenases in heterocystous N_2-fixing cyanobacteria**. *Biochem Biophys Res Comm* 2001, **283**:356−363.
7. Nakahara K, Yamamoto H, Miyake C, Yokota A: **Purification and characterization of class-I and class-II fructose-1,6-bisphosphate aldolases from the cyanobacterium *Synechocystis* sp PCC 6803**. *Plant Cell Physiol* 2003, **44**:326−333.
